# Cardiomyocyte precursors generated by direct reprogramming and molecular beacon selection attenuate ventricular remodeling after experimental myocardial infarction

**DOI:** 10.1186/s13287-023-03519-w

**Published:** 2023-10-15

**Authors:** Dipthi Bachamanda Somesh, Kristin Klose, Janita A. Maring, Désirée Kunkel, Karsten Jürchott, Stephanie I. Protze, Oliver Klein, Grit Nebrich, Matthias Becker, Ulrike Krüger, Timo Z. Nazari-Shafti, Volkmar Falk, Andreas Kurtz, Manfred Gossen, Christof Stamm

**Affiliations:** 1 BIH Center for Regenerative Therapies, Berlin Institute of Health at Charité – Universitätsmedizin Berlin, 13353 Berlin, Germany; 2 Berlin-Brandenburg School for Regenerative Therapies, Charité – Universitätsmedizin Berlin, 13353 Berlin, Germany; 3 Institute of Active Polymers, Helmholtz-Zentrum Hereon, 14513 Teltow, Germany; 4 Berlin-Brandenburg Center for Regenerative Therapies, 13353 Berlin, Germany; 5 Department of Cardiothoracic and Vascular Surgery, Deutsches Herzzentrum der Charité – Medical Heart Center of Charité and German Heart Institute Berlin, Augustenburger Platz 1, 13353 Berlin, Germany; 6 Cytometry Core Facility, Berlin Institute of Health at Charité – Universitätsmedizin Berlin, 13353 Berlin, Germany; 7 Charité – Universitätsmedizin Berlin, Institute for Medical Immunology, 13353 Berlin, Germany; 8 University Health Network, McEwen Stem Cell Institute, Toronto, ON M5G 1L7 Canada; 9 Department of Molecular Genetics, University of Toronto, Toronto, ON M5S 1A8 Canada; 10 BIH Imaging Mass Spectrometry Core Unit, Berlin Institute of Health at Charité – Universitätsmedizin Berlin, 13353 Berlin, Germany; 11 German Centre for Cardiovascular Research, Partner Site Berlin, 10785 Berlin, Germany; 12 Department of Health Sciences and Technology, ETH Zurich, 8092 Zurich, Switzerland

**Keywords:** Cell therapy, Direct reprogramming, Fibroblast, Induced cardiomyocyte precursor, Molecular beacon, Myocardial infarction, Transdifferentiation

## Abstract

**Background:**

Direct cardiac reprogramming is currently being investigated for the generation of cells with a true cardiomyocyte (CM) phenotype. Based on the original approach of cardiac transcription factor-induced reprogramming of fibroblasts into CM-like cells, various modifications of that strategy have been developed. However, they uniformly suffer from poor reprogramming efficacy and a lack of translational tools for target cell expansion and purification. Therefore, our group has developed a unique approach to generate proliferative cells with a pre-CM phenotype that can be expanded in vitro to yield substantial cell doses.

**Methods:**

Cardiac fibroblasts were reprogrammed toward CM fate using lentiviral transduction of cardiac transcriptions factors (GATA4, MEF2C, TBX5, and MYOCD). The resulting cellular phenotype was analyzed by RNA sequencing and immunocytology. Live target cells were purified based on intracellular CM marker expression using molecular beacon technology and fluorescence-activated cell sorting. CM commitment was assessed using 5-azacytidine-based differentiation assays and the therapeutic effect was evaluated in a mouse model of acute myocardial infarction using echocardiography and histology. The cellular secretome was analyzed using mass spectrometry.

**Results:**

We found that proliferative CM precursor-like cells were part of the phenotype spectrum arising during direct reprogramming of fibroblasts toward CMs. These induced CM precursors (iCMPs) expressed CPC- and CM-specific proteins and were selectable via hairpin-shaped oligonucleotide hybridization probes targeting Myh6/7-mRNA–expressing cells. After purification, iCMPs were capable of extensive expansion, with preserved phenotype when under ascorbic acid supplementation, and gave rise to CM-like cells with organized sarcomeres in differentiation assays. When transplanted into infarcted mouse hearts, iCMPs prevented CM loss, attenuated fibrotic scarring, and preserved ventricular function, which can in part be attributed to their substantial secretion of factors with documented beneficial effect on cardiac repair.

**Conclusions:**

Fibroblast reprogramming combined with molecular beacon-based cell selection yields an iCMP-like cell population with cardioprotective potential. Further studies are needed to elucidate mechanism-of-action and translational potential.

**Graphical Abstract:**

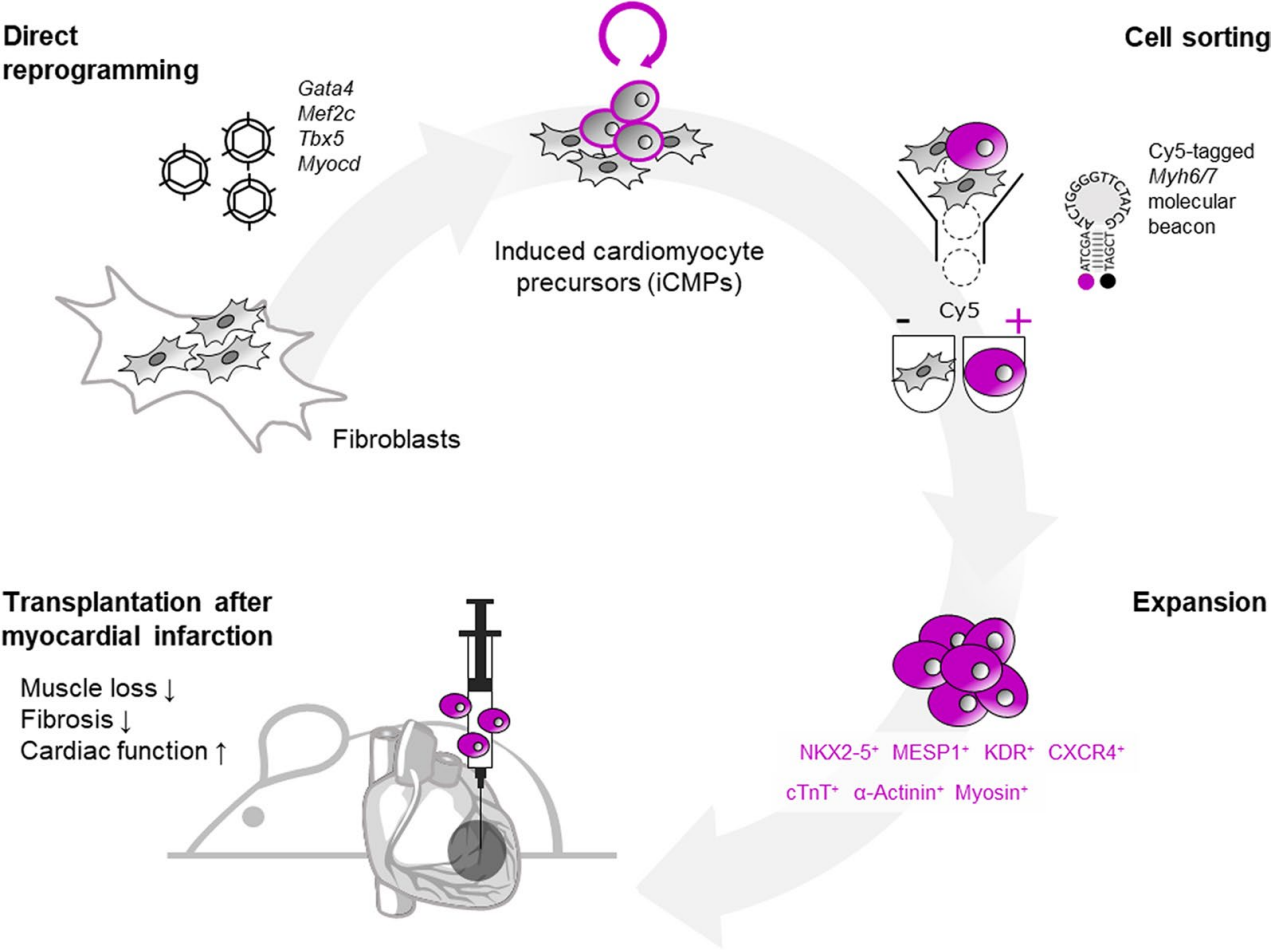

**Supplementary Information:**

The online version contains supplementary material available at 10.1186/s13287-023-03519-w.

## Background

Over the past two decades, numerous cardiac cell therapy approaches have been investigated for restoration of impaired left ventricular function (reviewed in [1]). In clinical trials, however, improvements in heart function after injection of somatic progenitor cells have not been clinically meaningful, if detectable at all (reviewed in [2–5], own experience [6–10]). There is now consensus that, apart from limited cell retention and survival, the cardiomyocyte (CM) (trans) differentiation inability of transplanted cells has been the main reason for unmet expectations (reviewed in [4, 11, 12]).

Therefore, research efforts have been redirected to cellular reprogramming technologies that enable the de novo generation of contractile cells. One such approach is the direct reprogramming of nonmyocytes into induced CM (iCM), either through the delivery of unique combinations of cardiomyogenesis-related transcription factors and microRNAs (direct conversion), or through transient exposure to pluripotency transcription factors followed by signaling-directed differentiation (primed conversion) (reviewed in [13]). In the work by Ieda et al. and Jayawardena et al., direct iCM conversion was accomplished with GATA4, MEF2C, and TBX5 [14], or with microRNAs 1, 133, 208, and 499 [15], respectively. Efe et al. demonstrated primed iCM conversion by transient exposure to OCT4, SOX2, KLF4, and MYC, followed by JAK‐STAT signaling inhibition and BMP4 treatment [16]. Based on these original approaches for direct reprogramming of fibroblasts into CM-like cells, various modifications of that strategy have been developed. However, they uniformly suffer from low reprogramming efficacy and a lack of translational tools for target cell expansion and purification, which poses a major challenge for the preparation of sufficient CM numbers required for cardiac cell therapy [13].

Therefore, reprogramming into proliferative cardiac progenitor cells (CPC) may have advantages over reprogramming into quiescent CMs, because limiting lineage conversion efficiencies may be compensated for by in vitro expansion [13]. Proliferative progenitor cells may also provide additional benefits in the setting of cardiac cell therapy after ischemic injury: (1) in vivo proliferation to compensate cell loss during delivery, (2) resilience to hostile ischemic environment (e.g., to hypoxia via anaerobic glycolysis (reviewed in [17])), (3) immunomodulation [18], (4) secretory activity to establish a favorable microenvironment [19], (5) migration into areas far from the injection site, and (6) CM differentiation at final destination.

Reprogramming of fibroblasts into expandable induced CPCs (iCPCs) with tripotent differentiation potential for CMs, endothelial cells, and smooth muscle cells has previously been reported by Lalit et al. and Zhang et al. [20, 21]. Such iCPC populations are usually heterogeneous pools of uni-, bi-, and tripotent cells of varying maturity. iCPC pool composition depends on the starting cell batch and is difficult to control, which leads to highly variable CM differentiation efficiencies as well as unpredictable and/or low CM yields. For therapeutic use, however, the target cell product should be well-defined and homogenous.

Given the lack of CM-precursor–specific surface markers, live cell purification relies on intracellular markers. Transgenic reporter systems are available but require additional, clinically unfavorable, genetic modifications. Alternatively, intracellular marker expression can be monitored using molecular beacons, transfectable, labeled hairpin oligonucleotides that hybridize with complementary mRNA and emit fluorescence after excitation, allowing of target cell purification in a clinically translatable fashion.

Here, we demonstrate a unique approach to generate proliferative cells with a pre-CM phenotype that can be expanded in vitro to yield substantial cell doses. Following transcription factor-induced reprogramming of cardiac fibroblasts using GATA4, MEF2C, TBX5, and MYOCD (GMTMy), we use molecular beacon technology to enrich the target cell population of induced CM precursors (iCMPs) based on *Myh6/7*-expression. iCMPs exhibit gene expression signatures of developing CMs and are capable differentiation into rod-shaped, sarcomere-forming CM-like cells. In a mouse model of acute myocardial infarction, intramyoardial iCMP transplantation exerts therapeutic effects, making iCMPs a promising off-the-shelf candidate for cardiac regenerative cell therapy.

## Methods

### Fibroblast culture

Primary neonatal C57BL/6 J mouse cardiac fibroblasts were purchased from PELOBIOTECH (cat. no. PB-C57-6049). For expansion, fibroblasts (5000/cm^2^) were plated into gelatin-coated (0.2 mg/ml, Sigma-Aldrich) vessels (CELLSTAR, Greiner Bio-One) and cultured in high glucose Dulbecco's modified eagle medium supplemented with heat-inactivated fetal bovine serum (10%) and penicillin/streptomycin (1%; 100 U/ml, 100 μg/ml) (standard medium, all Gibco) at 37 °C and 5% CO2 in a humified incubator. All experiments were performed with passage 2–5 cells.

### Cardiac cell isolation and culture

Single-cell suspensions of primary neonatal cardiac cells (CMs, endothelial cells, smooth muscle cells, etc.) were isolated from healthy neonatal (age, 2–4 days) C57BL/6 J wildtype and homozygous *Myh6*-*mCherry*–transgenic mice (strain, [B6;D2-Tg(Myh6*-mCherry)2Mik/J], The Jackson Laboratory, stock no. 021577, [22]) as described previously with minor modifications [23]. Collagenase B (Roche) was used for tissue digestion. CMs were not purified. Erythrocytes were removed using the 10X red blood cell lysis solution from Miltenyi Biotec. Before molecular beacon transfection, cardiac cells were plated (100,000/cm^2^) into fibronectin-coated (0.005 mg/ml, Sigma-Aldrich) vessels and cultured for 24 h.

### Cell lines

HEK293TN (System Biosciences, cat. no. LV900A-1) and HT1080 (ATCC, cat. no. CCL-121) cells were cultured in standard medium and used for lentivirus production or titration.

### Lentiviral plasmid cloning, lentivirus production, and lentiviral transduction efficiency

Human-immunodeficiency-virus-based lentiviral gene transfer plasmids encoding the murine cardiac transcription factors *Gata4*, *Mef2c*, *Tbx5*, or *Myocd* in combination with an enhanced green fluorescent protein (*eGFP*) transduction reporter and the *neo* resistance gene were cloned as described previously [24]. Control plasmids encoded only *eGFP*.

2nd-generation lentiviruses were produced in HEK293TN cells, concentrated, and biologically titrated in HT1080 cells as described previously [25]. Titers ranged from 67 to 330 million transducing units per ml. Infection of adherent cardiac fibroblasts with individual lentiviruses at a multiplicity of infection of 5 resulted in 89 ± 3% eGFP-positive cells (mean ± SD; Additional file 1: Fig. S1).

### Direct fibroblast reprogramming/transdifferentiation

**Fig. 1:**
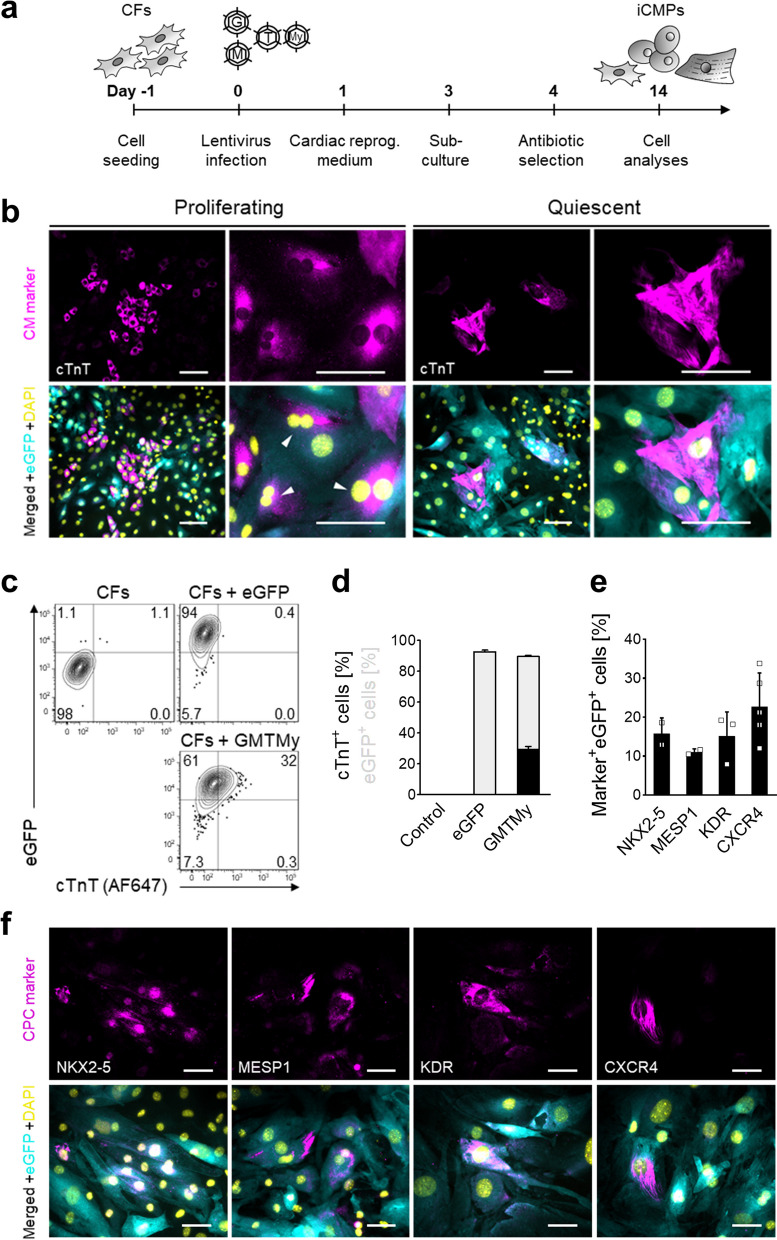
*GMTMy* overexpression directly reprograms cardiac fibroblasts into iCMPs. **a** Schematic outline of the protocol used to convert cardiac fibroblasts (CFs) toward CM lineage. **b** Immunofluorescent staining for CM marker cTnT (magenta) in combination with eGFP (cyan) 14 days after lentiviral *GMTMy* delivery reveals the coinduction of two subpopulations: (1) clusters of immature, proliferating cTnT-positive cells (left panel, white arrows point to mitotic nuclei); and (2) isolated, more mature, nonproliferative cTnT-positive cells (right panel). DAPI (yellow) marks cell nuclei. Scale bars, 100 µm. Single channel images are provided in Additional file 1: Fig. S3c. **c** Analysis of cTnT protein expression by flow cytometry on day 14 in selected experiments (in total *n* > 15 biologically independent experiments). Shown are contour plots (contour level 5%). **d** Quantification of cTnT expression. Stacked bar graph presents the proportion of cTnT- and eGFP-positive cells in (C) as mean ± SD (*n* = 2). **e** CPC marker expression in *GMTMy*-transduced cells on day 14 as determined by immunocytology and subsequent image-based quantification. Scatter plot with bar graph shows replicates and mean ± SD (*n* = 2–5). **f** Immunofluorescent staining for CPC markers (magenta) NKX2-5, MESP1, KDR, and CXCR4 in combination with eGFP (cyan) on day 14 indicates that GMTMy induces a cardiac progenitor signatures. DAPI (yellow) marks cell nuclei. Scale bars, 50 µm Figure 2

Cardiac fibroblasts were plated (5000/cm^2^) in standard medium into gelatin-coated vessels. The next day, fibroblasts were infected with the GMTMy-lentivirus-cocktail at a multiplicity of infection of 5. After 24 h, standard medium was replaced with cardiac reprogramming medium, composed of low glucose Dulbecco's modified eagle medium and M199 (3:1) and supplemented with heat-inactivated foetal bovine serum (10%) and penicillin/streptomycin (1%). Control cells were infected with eGFP-lentiviruses at a multiplicity of infection of 20. On day 3 after lentivirus infection, cells were sub-cultured (5,000/cm^2^). From day 4 onwards, cells were subjected to geneticin (1 mg/ml; Fisher Scientific) selection. Thereafter, medium was changed every other day until day 14 (see reprogramming timeline in Fig. 1a).

### Immunocytology of adherent cells and fluorescence microscopy

At indicated points in time, cells were fixed in paraformaldehyde (paraformaldehyde, 4%, Carl Roth) and incubated in permeabilization and blocking buffer (0.25% Triton X 100 and 10% secondary antibody-matched serum in Dulbecco's phosphate-buffered saline (DPBS)). Thereafter, cells were incubated with primary antibodies overnight at 4 °C and with secondary antibodies for 2 h at room temperature in the dark (Additional file 1: Table S1). Cells were stained with DAPI (Invitrogen) before images were acquired using the Axio Observer Z1 fluorescence microscope equipped with AxioVision software (version 4.9.1.0) (both Carl Zeiss). Image-based quantification of CPC marker expression was performed in ImageJ (version 1.53a, NIH) using 20X images and the Cell Counter plugin.

### Immunocytology of harvested cells and flow cytometry

At indicated points in time, cells were harvested, fixed in paraformaldehyde (4%), and incubated in permeabilization and blocking buffer. Thereafter, cells were incubated with primary antibodies for 1 h at room temperature and with secondary antibodies for 30 min at room temperature in the dark (Additional file 1: Table S1). Cells were resuspended in cell sorting buffer before analysis on a MACSQuant flow cytometer equipped with MACSQuantify software (both Miltenyi Biotech). Data were analyzed with FlowJo software (versions 7.6.5 or 10; BD Biosciences). A representative gating strategy is provided in Additional file 1: Fig. S4.

### Molecular beacons and iCMP sorting

Molecular beacons are single-stranded oligonucleotides that form a stem-loop structure in the absence of their complementary target sequence. In the presence of their target sequence, the probes undergo conformational changes to hybridize to their target, resulting in opening of the stem, separation of fluorophore and quencher, and finally fluorescence after excitation. The following molecular beacons were used for analysis of *Myh6/7* mRNA expression and iCMP sorting: (1) negative control (probe degradation control, random sequence without match in mouse genome, cyanine 5 (Cy5) and Black Hole Quencher 2 (BHQ2) label at 5’- and 3’-end, respectively); (2) delivery control (transfection control, random sequence, Cy5-Cy5 label); and (3) *Myh6/7* beacons (specific target sequence, Cy5-BHQ2 label). Molecular beacon sequences have been published previously [26]. The beacons were synthesized as DNA probes by Microsynth Seqlab. 100 µM stock solutions were prepared by resuspension in TE buffer (pH 7.0, Invitrogen) and stored at − 20 °C.

Optimal conditions for the molecular beacon transfection using the X-tremeGENE HP DNA Transfection Reagent (lipid, Roche) were determined in *GMTMy*-transduced cardiac fibroblasts using the delivery control beacon (Additional file 1: Fig. S5a). CM specificity was confirmed in *Myh6*-*mCherry*–transgenic cardiac cells using the *Myh6/7* beacon (Additional file 1: Fig. S5b).

After GMTMy reprogramming of fibroblasts, live cells were sorted on day 14 after lentivirus infection according to the following protocol: *Myh6/7* beacon stock solution (1 µl), transfection reagent (4 µl), and cardiac reprogramming medium (95 µl) were combined and preincubated for 20 min. The transfection mix was combined with additional medium (300 µl), added to adherent cells (400 µl/cm^2^), and incubated for 4 h at 37 °C. Control cells were transfected with the negative control probe. Transfected cells were harvested and resuspended in cell sorting buffer (2% bovine serum albumin, 2 mM EDTA in DPBS; Carl Roth).

*Myh6/7* mRNA expression analysis and iCMP sorting were performed by the BIH Cytometry Core Facility on a BD FACSAria II SORP, configured with 4 lasers (violet, blue, yellow-green, red), and equipped with FACSDiva software (versions 6.1.3 or 8.0.2) (both BD Biosciences). Representative gating strategies are provided in Additional file 1: Fig. S5a, c. Data were analyzed with FlowJo software. Sorted *Myh6/7*-Cy5–positive and *Myh6/7*-Cy5–negative cell samples were reanalyzed using the same sorter, the same gates, and the same settings as used during sorting. Enrichment of *Myh6/7*-Cy5–positive iCMPs was analyzed by immunocytological staining for CM markers. Purified iCMPs were plated (10,000/cm^2^) in cardiac reprogramming medium for subsequent analyses.

### iCMP expansion and phenotype maintenance

For expansion, purified iCMPs were plated (5000/cm^2^) into gelatin-coated vessels and maintained in cardiac reprogramming medium with supplements (see below) for 27 days. Every 3–4 days, at ~ 80% confluency, iCMPs were sub-cultured (5000/cm^2^). Viable cell counts were determined using trypan blue (Sigma-Aldrich) and cumulative population doubling levels (PDLs) were calculated using the following formular: PDL_harvested cells_ = 3.32[log(count cell harvest)–log(cell number plating)] + PDL_plated cells_. During expansion, cell morphology and *Myh6/7* expression were assessed regularly by microscopy and flow cytometry (see above), respectively.

iCMP phenotype maintenance was examined in basic cardiac reprogramming medium or in the presence of various supplements: (1) geneticin (0.5 mg/ml); (2) ascorbic acid (250 µg/ml, FUJIFILM Wako Chemicals Europe, cat. no. 13–19,641); (3) FGF2 (2 ng/ml) and VEGFA (5 ng/ml) (both PeproTech); (4) FGF2 (2 ng/ml), VEGFA (5 ng/ml), and BMP4 (20 ng/ml; R & D Systems); and (5) FGF2 (2 ng/ml) and BMP4 (20 ng/ml) [27]. Media were changed every other day.

### RNA isolation, RNA sequencing, and bioinformatics analyses

Total RNA was isolated from duplicate samples of purified iCMPs, starting cardiac fibroblasts, and mouse adult left ventricular heart tissue using the Qiagen RNeasy Mini Kit (Qiagen GmbH). Genomic DNA was removed with DNAse I (Sigma-Aldrich). RNA quality and quantity were assessed on a 2100 Bioanalyzer System using the RNA 6000 Nano Kit (both Agilent Technologies). For RNA sequencing, poly-(A)-selection was performed with 500 ng total RNA using the NEBNext Poly(A) mRNA Magnetic Isolation Module (NEB). RNA libraries were prepared using the NEBNext Ultra RNA Library Prep Kit for Illumina (NEB). Library preparation success was confirmed by analyzing the fragment size distribution on a 2100 Bioanalyzer using the DNA 1000 Kit (Agilent Technologies). Library concentration was determined using Qubit dsDNA BR Assay Kit and Qubit 3.0 (Thermo Fisher Scientific). After equimolar pooling, RNA samples were sequenced using a HiSeq 1500 System with Rapid Mode chemistry v2 (50 cycles, single-read; Illumina).

For RNA sequencing data analysis, sequence reads were demultiplexed using bcl2fastq2 (version 2.18, Illumina) and fastq file quality was assessed using fastqc (version 0.11.7, Bioinformatics Group at the Babraham Institute). Residual adapter sequences and low-quality reads were trimmed using AdapterRemoval (2.2.2) [28]. Reads were aligned to the mm10 (GRCm38.82) version of the mouse genome using tophat (version 2.1.0) [29] and bowtie2 (version 2.2.5) [30]. Counts per gene were calculated as the sum of all mapped reads within a gene region. Raw counts of protein-coding genes were normalized and variance stabilizing transformed using DESeq2 (1.28.1) [31] in R (A language and environment for statistical computing. R Foundation for Statistical Computing, Vienna, Austria. URL https://www.R-project.org/; 4.0.2). Variances across all samples were determined for each gene and 1,000 genes with the highest variances were selected for unsupervised analysis. Principal component analysis of the 1000 top-variably expressed genes was done with the prcomp() function in R. Differentially expressed genes were identified in pairwise comparisons between treatment groups using a negative binominal model provided by DESeq2. Bonferroni-corrected *P* < 0.05 and a minimal absolute log2 fold change of 1 were used to select significant results. K-means clustering of differentially expressed genes was based on Euclidean distances and performed using scaled data and the in-built kmeans() function in R with 100 randomly defined start sets of 6 clusters. Overrepresentation analysis of Differentially expressed genes in terms of the gene ontology system was done using the topGO package (Adrian Alexa and Jörg Rahnenführer (2016). topGO: Enrichment Analysis for Gene Ontology. R package version 2.32.0.) in R using classical Fisher tests based on hypergeometric distributions to asses significance of enrichment. Due to the high redundancy of the gene ontology system, raw P values were not adjusted for multiple testing. Heatmaps were generated using scaled data. The k-means heatmap was generated in R using the in-built heatmap function; the selected genes heatmaps were generated in GraphPad Prism (version 8 and 9, GraphPad Software). RNA sequencing data sets have been archived in the NCBI Gene Expression Omnibus data repository (GSE159315).

### Reverse transcription-quantitative PCR

For reverse transcription-quantitative PCR, 100 ng RNA were random hexamer primed and reverse transcribed into cDNA using the SuperScript III First-Strand Synthesis System (Invitrogen). PCR reactions (8 µl each) were prepared by combining cDNA samples (final, 0.125 ng/µl reaction) with gene-specific primers (final, 0.5 µM), and the 2X Power SYBR Green PCR Master Mix (final, 1X; Applied Biosystems). Quantitative PCR was performed on the Quant Studio 6 Flex Real-Time PCR System (Applied Biosystems) with the following PCR program: initial denaturation (95 °C, 10 min), annealing and extension (50 cycles: 95 °C, 15 s; 60 °C, 30 s; 72 °C 30 s), melting curve program. Relative gene expression levels were quantified using the ∆ct-method, were ct is the threshold/quantification cycle and ∆ct = ct(geometric mean *Rpl13a*, *B2m*)-ct(gene of interest). *B2m* (beta-2-microglobulin) and *Rpl13a* (ribosomal protein L13a) served as reference genes. PCR amplification efficiencies were 1.9–2. PCR primers were designed using Primer-BLAST(NCBI) [32] and Primer3 (open software development project) [33], or obtained via PrimerBank [34] (*B2m*, ID 31981890a1; *Tnnt2*, ID 6755843a1) (Additional file 1: Table S2). Primers were synthesized by Eurofins Genomics.

### Cardiac differentiation of iCMPs

To induce cardiac differentiation, iCMPs were stimulated with 5-azacytidine (Sigma-Aldrich), TGFB1 (PeproTech), and ascorbic acid, and subsequently cultured for 20 days in differentiation medium as described previously [35] (see differentiation timeline in Fig. 04a). Cardiac differentiation was initiated 2–3 days after sorting and plating (5,000/cm^2^) into gelatin-coated vessels. During differentiation, cell morphology, cardiovascular protein expression, and spontaneous contractility were regularly assessed by microscopy and immunocytology.

### Mouse myocardial infarction model, cell transplantation

103 male C57BL/6 J mice (Charles River), 10–12 weeks old and 20–30 g in weight, were used to evaluate the therapeutic effect of iCMP transplantation after myocardial infarction in 2 study arms: (1) serial measurement of heart function by echocardiography and determination of scar size after 6 weeks; and (2) iCMP location by immunohistology 2 weeks after transplantation. The week-6 group size (n_SHAM_ = 10, n_myocardial infarction groups_ = 23) was calculated under consideration of mortality rates and cardiac parameters of previous experiments (fractional shortening) as well as the planned statistical analysis. The week-2 group sizes (n_myocardial infarction groups_ = 8) was estimated owing to the explorative aim of iCMP location. On each operation day, animals were randomly assigned to one of four groups: SHAM control, vehicle control, noninduced cell control, or iCMP group.

Animals were anesthetized by intraperitoneal injection of narcosis mix (150 µl; fentanyl (0.08 mg/kg, Rotexmedica), midazolam (8 mg/kg, Ratiopharm), and medetomidine (0.4 mg/kg, cp-pharma)) and subsequently intubated. Permanent left anterior descending artery ligation was performed as described previously [36]. Phosphate-buffered saline (DPBS, vehicle control, *n* = 31) or purified *eGFP*-transduced cardiac fibroblasts (CFs^eGFP^, noninduced cell control, 5 × 10^5^ cells, *n* = 31), or purified iCMPs (5 × 10^5^ cells, *n* = 31) were injected at 2 sites of the lateral infarct border zone (5 µl each). Animals were awakened by intraperitoneal injection of antagonist mixture (100 µl; flumazenil (0.3 mg/kg, hameln), atipamezole (1.7 mg/kg; cp-pharma), and buprenorphine (0.1 mg/kg; Indivior UK Limited)), along with a subcutaneous injection of carprofen (100 µl, 5 mg/kg, Zoetis). All surgical procedures were carried out under aseptic conditions by a single investigator that was blinded for intervention. For sustained pain relief, animals were given metamizole (Ratiopharm) in drinking water (7 mg/ml in 5% glucose, B. Braun) daily for 5 days. Animals were monitored regularly for 2–6 weeks using model-specific score sheets and housed in individually ventilated cages (caging density 2–5) under specific pathogen-free conditions at the Charité Research Facility for Experimental Medicine. Animals were sacrificed by cardiac puncture. SHAM animals (*n* = 10) underwent the same procedures, except for left anterior descending artery ligation and intervention. Data were excluded when: (1) myocardial blanching was not observed during operation and absent infarction was corroborated by echocardiography and/or histological analysis; or (2) follow-up was not available. Overall mortality was 35%, including intra-, peri-, and postoperative deaths. Accordingly, group sizes in week 2/6 were: n_SHAM_ = 0/10, n_DPBS_ = 5/12, n_CFeGFP_ = 5/14, n_iCMP_ = 6/13.

### Transthoracic echocardiography

Transthoracic echocardiography was performed 2, 4, and 6 weeks after myocardial infarction using the Vevo 2100 Imaging System equipped with a MS550D transducer and Vevo LAB analysis software (version 03.01.00, all FUJIFILM VisualSonics). Anesthesia was induced with 3–4% isoflurane (AbbVie) and maintained with 1–2% isoflurane (O_2_ = 2 l/min). For all animals, static and cine loop images were acquired from the left ventricle in parasternal long axis and short axis views during electrocardiogram-gated kilohertz visualization, B-Mode, and M-Mode scanning. Images were analyzed using the “Left Ventricle Trace” tool of Vevo LAB. For each animal, measurements from 3 cardiac cycles were averaged for subsequent analysis. Myocardial-infarction–induced anatomical irregularities occasionally interfered with ultrasound imaging and analysis and resulted in missing values. The range of values for each parameter was n_SHAM_ = 7–10, n_DPBS_ = 8–11, n_CFeGFP_ = 10–14, n_iCMP_ = 9–13. All analyses were performed by a single investigator that was blinded for intervention.

### Histology and immunohistology

2 and 6 weeks after myocardial infarction and treatment, hearts were excised from sacrificed animals, dehydrated in sucrose solution (15%, Sigma-Aldrich) overnight at 4 °C, and frozen in Tissue-Tek optimal cutting temperature compound embedding medium (A. Hartenstein). Transverse sections of 7 μm thickness were cut from the apex to the ligation site, with 280 µm intervals after every 80 sections, and mounted onto glass slides for histological analysis.

For scar size quantification, cryosections were fixed in paraformaldehyde (4%) and Masson trichrome staining (Sigma-Aldrich) was performed according to manufacturer’s instructions. Stained sections were imaged using the NanoZoomer-SQ slide scanner (Hamamatsu Photonics). The scarred and total left ventricular areas were quantified using ImageJ. Infarct scar size was calculated by dividing scarred tissue areas by total left ventricular areas and multiplying by 100 (n_DPBS_ = 7, n_CFeGFP_ = 7, n_iCMP_ = 9).

For immunohistology, cryosections were fixed in paraformaldehyde (4%) and incubated in permeabilization and blocking buffer. Thereafter, cryosections were incubated with primary antibodies overnight at 4 °C and with secondary antibodies for 2 h at room temperature in the dark (Additional file 1: Table S1). Sections were mounted in Fluoromount-G with DAPI (Invitrogen) before images were acquired using the Axio Observer Z1 fluorescence microscope equipped with AxioVision software or the TCS SP8 STED confocal microscope equipped with LAS X LS software (both Leica). Image acquisition and analysis was performed in a blinded manner.

### Mass spectrometry and paracrine profiling

Proteomic profiling of medium supernatants from iCMPs and CFs, cultured under serum-free normoxic (21% O_2_, 1 g/l glucose) or ischemic (1% O_2_, no glucose) conditions for 24 h or 72 h, were analyzed by liquid chromatography/electron spray ionization mass spectrometry (LC/ESI–MS). Medium supernatants were harvested, centrifuged for 10 min at 1000 g and 4 °C to remove cell debris, and frozen at -80 °C until further analysis. When all samples were collected, supernatants were thawed and transferred to amico filters (10kDA cut off, Merck) followed by an overnight trypsin digestion (12 µg trypsin in 50 mM ammonium bicarbonate; Promega) at 37 °C. Thereafter, peptide samples were extracted with trifluoroacetic acid (0.1%; Fluka) and measured by UPLC-ESI-QTOF mass spectrometer (Dionex Ultimate 3000, ThermoFisher; Impact II, bruker daltonics). Mass spectra were analyzed using the SwissProt database with MASCOT software (version number 2.2, Matrix Science). The analysis parameters were as follows: (i) taxonomy: Mus musculus (Mouse); (ii) proteolytic enzyme: trypsin; (iii) maximum of accepted missed cleavages: 1; (iv) mass value: monoisotopic; (v) peptide mass tolerance 10 ppm; (vi) fragment mass tolerance: 0.05 Da; and vii) variable modifications: oxidation. Identified proteins were considered for further analysis if scores corresponded to *p* < 0.05 and if at least two samples showed at least one detected peptide. Protein interaction networks were visualized and functional enrichment analyses were performed using the STRING database (version 11.5, http://string-db.org).

### Statistical analysis

Data were analyzed and graphs generated using GraphPad Prism. Data are presented as mean ± SD (in vitro data) or mean ± SEM (in vivo data). Repeated measures cardiac function data were analyzed by fitting a mixed-effects model to the data instead of performing a repeated measures ANOVA because it can handle randomly missing values. The GraphPad Prism model uses a compound symmetry covariance matrix and is fit using Restricted Maximum Likelihood (REML). Sphericity (equal variability of differences) was not assumed, instead the Greenhouse–Geisser correction was used. Multiple comparisons between group means were performed at individual measurement times and corrected for by statistical hypothesis testing using Holm-Sidak's post-hoc tests. Change data were analyzed using an ordinary one-way ANOVA after the D'Agostino & Pearson normality test was passed and the Bartlett’s test did not reveal significant different variances. Parametric test statistics are reported as [F(DFn, DFd) = *F*-value, *P* = *P*-value], where F corresponds to the F-statistic and DFn or DFd correspond to the degree of freedom nominator or degree of freedom denominator, respectively. Scar size data were analyzed using the Kruskal–Wallis test followed by Dunn’s post-hoc test to determine statistical significance of intergroup differences. The nonparametric test was chosen because the assumption of equal variances as determined using Bartlett’s test was violated, whereas the Shapiro–Wilk normality test was passed. P-values smaller than 0.05 were considered statistically significant.

## Results

### GMTMy directly reprograms fibroblasts into proliferative cTnT-expressing cells

To initiate direct reprogramming toward CMs, we overexpressed the well-known cardioinductive transcription factors GATA4, MEF2C, TBX5, and MYOCD in neonatal cardiac fibroblasts (Fig. 1a, Additional file 1: Fig. S2) [13]. Overexpression of *GMTMy* in combination with *eGFP* as control for gene integration was accomplished by lentiviral transduction.

Three days after lentivirus infection, nuclear GMTMy expression was detected by immunocytology in eGFP-labeled cardiac fibroblasts (Additional file 1: Fig. S3a). On day 14 after *GMTMy* delivery, a significant portion of the transduced cells expressed multiple CM-specific proteins, namely cardiac muscle troponin T (cTnT), α-actinin, and myosin, indicating the beginning change of cell identity (Additional file 1: Fig. S3b, Fig. 1b). Flow cytometric quantification revealed that 29.4 ± 2.0% of the analyzed cells expressed cTnT (mean ± SD; Fig. 1c,d). Overall, the early reprogramming yield determined on day 14 amounted to 10 cTnT-positive cells for each initially seeded fibroblast.

Interestingly, immunofluorescent staining of adherent cells revealed that the majority of cTnT-positive cells appeared immature as indicated by scattered cTnT expression patterns, small size, round shape, cluster formation, and mitotic activity, implying proliferation ability (Fig. 1b, Additional file 1: Fig. S3c). More mature cTnT-positive cells that showed an advanced reprogramming degree as indicated by partially striated cTnT expression patterns, large size, polygonal shape, single appearance, and mitotic quiescence were less frequently observed (Fig. 1b, Additional file 1: Fig. S3c).

To examine a putative progenitor state of our immature cTnT-positive cells, we analyzed the expression of a panel of well-known intracellular and surface cardiac progenitor cell (CPC) markers by immunocytology on day 14. Although we did not detect ISL1 (Insulin gene enhancer protein ISL-1) and c-Kit (mast/stem cell growth factor receptor Kit), we found that reprogrammed cells expressed NKX2-5 (homeobox protein NK-2 homolog E, 15.8 ± 4.0%), MESP1 (mesoderm posterior protein 1, 11.1 ± 0.8%), KDR (kinase insert domain receptor, 15 ± 6.3%), and CXCR4 (C-X-C chemokine receptor type 4, 22.8 ± 8.6%) (mean ± SD; Fig. 1e,f). These results show that GMTMy reprogramming turns a fraction of fibroblasts into proliferative, CPC- as well as CM-marker–expressing cells, which we term induced CM precursors (iCMPs).

### Live *Myh6/7*-expressing iCMPs can be purified using molecular beacons and expanded with stable phenotype

**Fig. 2:**
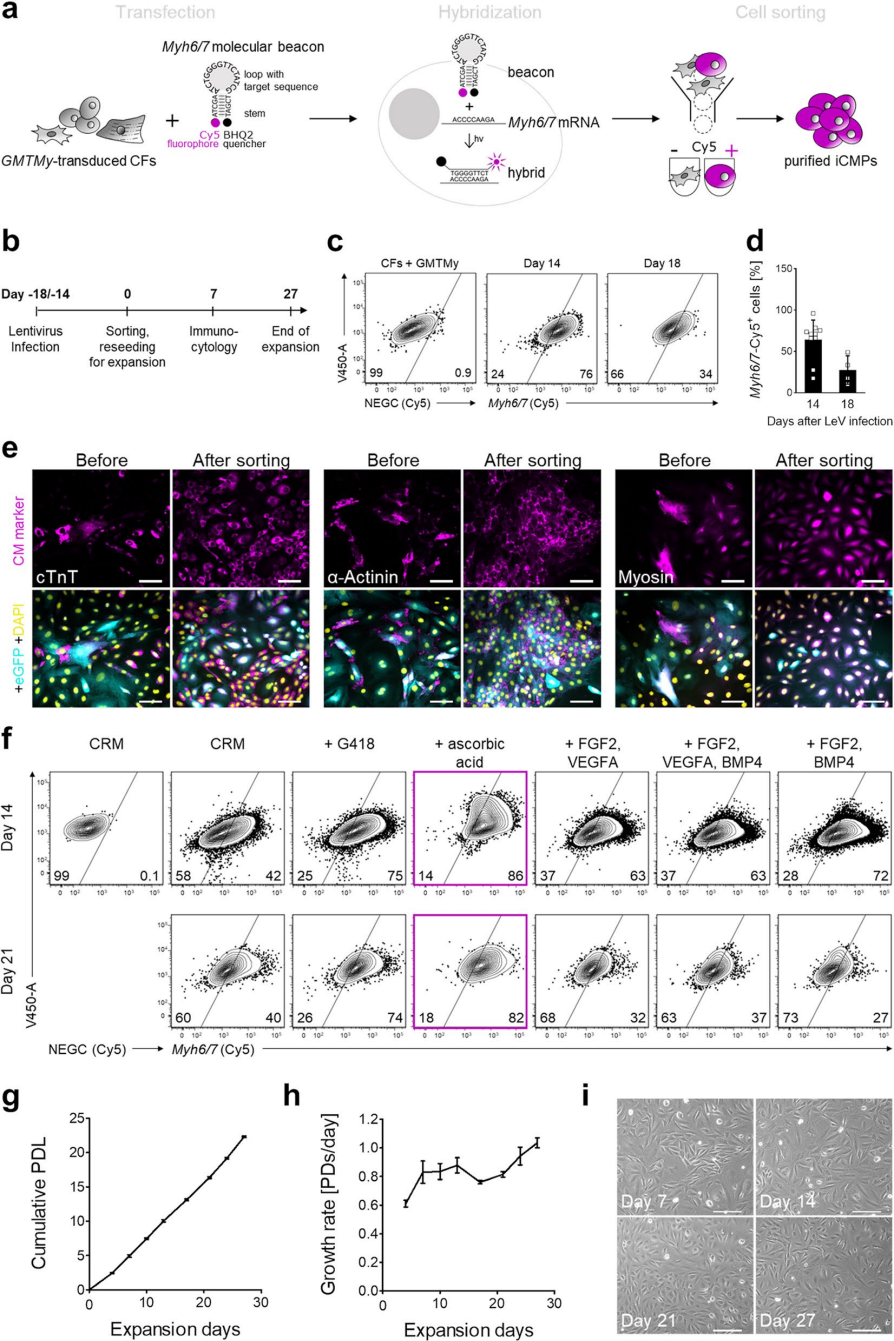
iCMPs are suitable for *Myh6/7*-molecular-beacon-based selection and capable of long-term propagation. **a** *Myh6/7* molecular beacon principle used to purify iCMPs. (Additional file 1: Fig. S5 for gating strategies) **b** Schematic outline of the experimental timeline. **c** Analysis of *Myh6/7* mRNA expression in reprogrammed cells by flow cytometry using Cy5-labeled negative control (NEGC, no sequence match, Cy5-BHQ2 label) and *Myh6/7* probes (sequence match, Cy5-BHQ2 label) on day 14 or 18 after *GMTMy* transduction. Shown are contour plots (contour level 5%). **d** Scatter plot with bar graph presents the proportion of Cy5-positive cells in (**c**) as mean ± SD (day 14/18, *n* = 10/4). **e** Immunofluorescent staining for cTnT, α-actinin, and myosin (magenta) in combination with eGFP (cyan) in *GMTMy*-transduced cells immediately before and 7 days after fluorescence-activated cell sorting demonstrates successful iCMP enrichment. DAPI (yellow) marks cell nuclei. Scale bars, 100 µm. **f** iCMP phenotype stability is dependent on expansion medium composition. Flow cytometric analysis of *Myh6/7*-based Cy5 fluorescence 14 and 21 days after expansion of purified iCMPs in media containing the respective supplements. **g,h** Cumulative population doubling levels (PDLs) (**g**) and growth rate as population doublings (PDs) per day **h** of iCMPs during expansion after sorting. Proliferation data were recorded for 27 days. Line graphs show mean ± SD (*n* = 4). **i** Brightfield images taken at indicated points in time during expansion show maintained iCMP morphology. Scale bars, 100 µm Figure 3

Because GMTMy reprogramming resulted in a heterogenous cell population, we next purified iCMPs for subsequent analyses. To ensure enrichment of CM-primed cells, we sorted iCMPs based on intracellular *Myh6/7* mRNA expression, an indicator for beginning CM fate specification (Fig. 2a, b). We chose an intracellular mRNA-based method because no CM-precursor–specific surface marker is available to date. To label *Myh6/7*-mRNA–expressing cells, reprogramming cultures were transfected with molecular beacons and subsequently underwent fluorescence-activated cell sorting. Molecular beacons are oligonucleotides, labeled with a fluorophore and a quencher, here Cy5 and BHQ2. In the absence of complementary mRNA, molecular beacons assume hairpin conformation with quenched fluorescence. After transfection of cells and hybridization to target mRNA, hairpins open, and beacons fluoresce after excitation, enabling cell sorting without clinically unfavorable genetic modifications. To optimize iCMP yield, *Myh6/7* mRNA expression was analyzed at different times, 14 and 18 days after reprogramming initiation. The highest yield of 64.4 ± 23.7% *Myh6/7*-expressing cells was achieved on day 14 (mean ± SD; Fig. 2c, d). After beacon-based cell sorting, target cell enrichment was confirmed by immunocytology for cTnT, α-actinin, and myosin (Fig. 2e). Hereafter, iCMP sorting was always performed on day 14.

We next sought to investigate iCMP proliferation capacity and phenotype stability. iCMPs actively proliferated, when cultured in reprogramming medium. However, the proportion of *Myh6/7*-positive iCMPs decreased markedly within 21 days after sorting to 40% of total cells, as determined by flow cytometry (Fig. 2f). Assuming that cardiac fibroblast overgrowth or iCMP dedifferentiation may be possible reasons, we either used geneticin antibiotic selection (with the *neo* resistance gene provided by the lentiviral vector) or supplemented the basal medium with one of the following CM-lineage–supporting factors or factor combinations to preserve iCMP phenotype: (1) ascorbic acid; (2) FGF2 + VEGFA, (3) FGF2 + VEGFA + BMP4; and (4) FGF2 + BMP4. Ascorbic acid was most effective in preserving iCMP phenotype, maintaining the percentage of *Myh6/7*-positive cells after 14 and 21 days at 86% and 82%, respectively (Fig. 2f). In all other media the percentage of *Myh6/7*-positive cells markedly decreased, in particular after 21 days of expansion. Finally, long-term expansion of iCMPs was initiated after iCMP sorting by serial passaging. During the expansion period of 27 days and 8 passages, iCMPs grew steadily with an average growth rate of 0.84 ± 0.13 population doublings per day, yielding an extrapolated number of 5.6 × 10^9^ cells per 10^3^ initially plated cells, corresponding to a six-million–fold increase (Fig. 2g, h). Cell morphology was also preserved during expansion, supporting the notion of iCMP phenotype stability (Fig. 2i). Taken together, these findings demonstrate that iCMPs can be enriched based on *Myh6/7* mRNA expression using molecular beacon technology and that ascorbic acid supplementation preserves molecular and morphological iCMP features during long-term expansion, rendering iCMPs suitable for large-scale cell production.

### iCMPs display global gene expression signatures of developing CMs

**Fig. 3:**
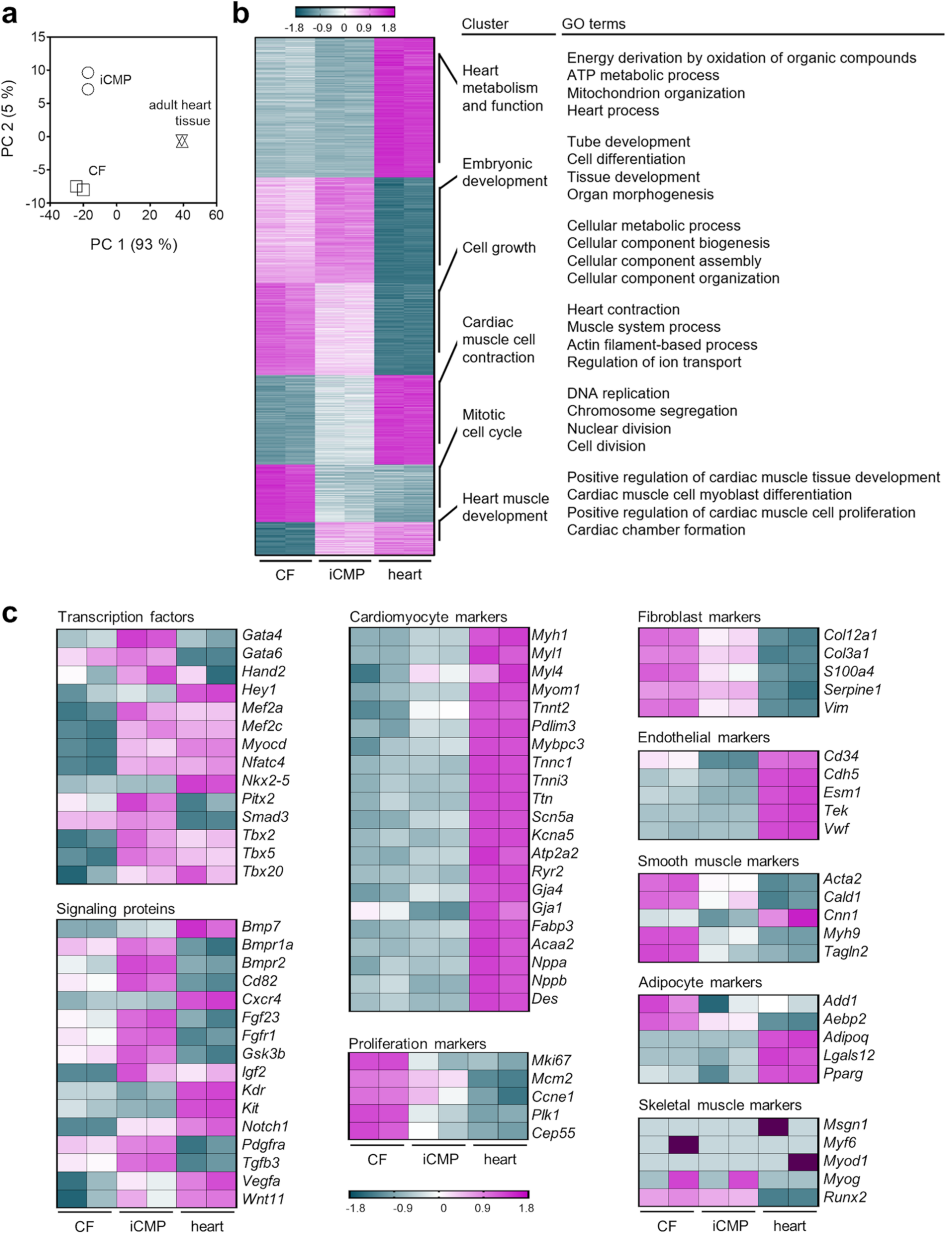
Global transcriptome analysis reveals suppression of fibroblast and induction of CM precursor signatures in iCMPs. **a** Principal component analysis of gene expression data generated by RNA sequencing from three sample groups: (1) purified iCMPs; (2) starting cardiac fibroblasts (CFs); and (3) adult heart (*n* = 2 for each sample group). The 2D principal component analysis plot presents the intra- and intergroup relationships by showing the first two principal components (PCs) that account for the majority (98%) of variation between samples. **b** K-means clustering and functional overrepresentation analysis of significant differentially expressed genes. Results are depicted as heatmap with accompanying table listing four gene ontology (GO) terms, selected from the top 40 terms with significant gene enrichment, for each of the six k-means clusters. **c** Analysis of selected marker genes associated with CM development, CM identity, proliferation, fibroblast identity, as well as related and competing cell lineages. Results are depicted as heatmaps. Heatmap columns represent replicate samples, heatmap rows represent single genes, the Z-score color scale indicates relative gene expression levels for each row (magenta, higher expression, Z-score > 0; cyan, lower expression, Z-score < 0; dark magenta, outside the defined range) Figure 4

To characterize iCMPs in more detail, we examined the global transcriptome of purified iCMPs in comparison with starting fibroblasts and adult heart tissue by RNA sequencing with subsequent bioinformatics analysis (please refer to the *Methods* section for details). Principal component analysis of gene expression data revealed that iCMPs were substantially different from cardiac fibroblasts, but did not yet cluster with heart (Fig. 3a). Similarly, differential gene expression analysis indicated a smaller difference between iCMPs and heart (6197 differentially expressed genes) and a larger difference between fibroblasts and heart (6628 differentially expressed genes). To identify clusters of differentially expressed genes and biological processes associated with them, we next performed k-means clustering and functional overrepresentation analysis (Fig. 3b). Expression of genes related to embryonic development, heart muscle development, and cardiac muscle cell contraction were upregulated in iCMPs compared to fibroblasts, indicating beginning CM conversion. However, compared to heart, expression levels were lower, suggesting an intermediate cardiogenic state. By contrast, expression of genes related to mitotic cell cycle, cell growth as well as heart metabolism and function were downregulated in iCMPs compared to cardiac fibroblasts, suggesting a reprogramming-induced proliferation decrease and CM precursor rather than CM conversion. Although cell cycle gene expression was suppressed in iCMPs compared to fibroblasts, expression was still higher compared to heart, which is in line with our observation of continuous iCMP proliferation after sorting (Fig. 2f–i).

To determine the progress in cell fate transition, we evaluated the expression of selected genes known to be associated with CM fate commitment, CM identity, proliferation, fibroblast identity, and unintended cell fates (Fig. 3c). We found that enforced *GMTMy* expression induces the expression of additional cardiac transcription factors and signaling proteins involved in CM development (e.g., *Hand2*, *Tbx20*, *Igf2, Vegfa*, *Wnt11*) as well as a subset of structural and functional markers of mature CM (e.g., *Myl4*, *Tnnt2*, *Mybpc3*, *Gja4*) indicating the activation of CM gene regulatory networks. Fibroblast and proliferation gene expression were reduced (e.g., *Col3a1*, *S100a4*, *Vim*, *Mki67*, *Mcm2*). GMTMy did not induce the expression of markers common to other cardiovascular cell fates (endothelial cells, smooth muscle cells), competing lineages (adipocytes) [13], or noncardiac muscle (skeletal muscle). To validate RNA sequencing findings, expression patterns for a subset of genes were studied using reverse transcription-quantitative PCR, revealing similar results (Additional file 1: Fig. S6). In summary, these analyses suggest that iCMPs possess transcriptional signatures that are different from cardiac fibroblasts and similar to CM precursor cells.

### iCMPs differentiate into sarcomere-forming CM-like cells

**Fig. 4:**
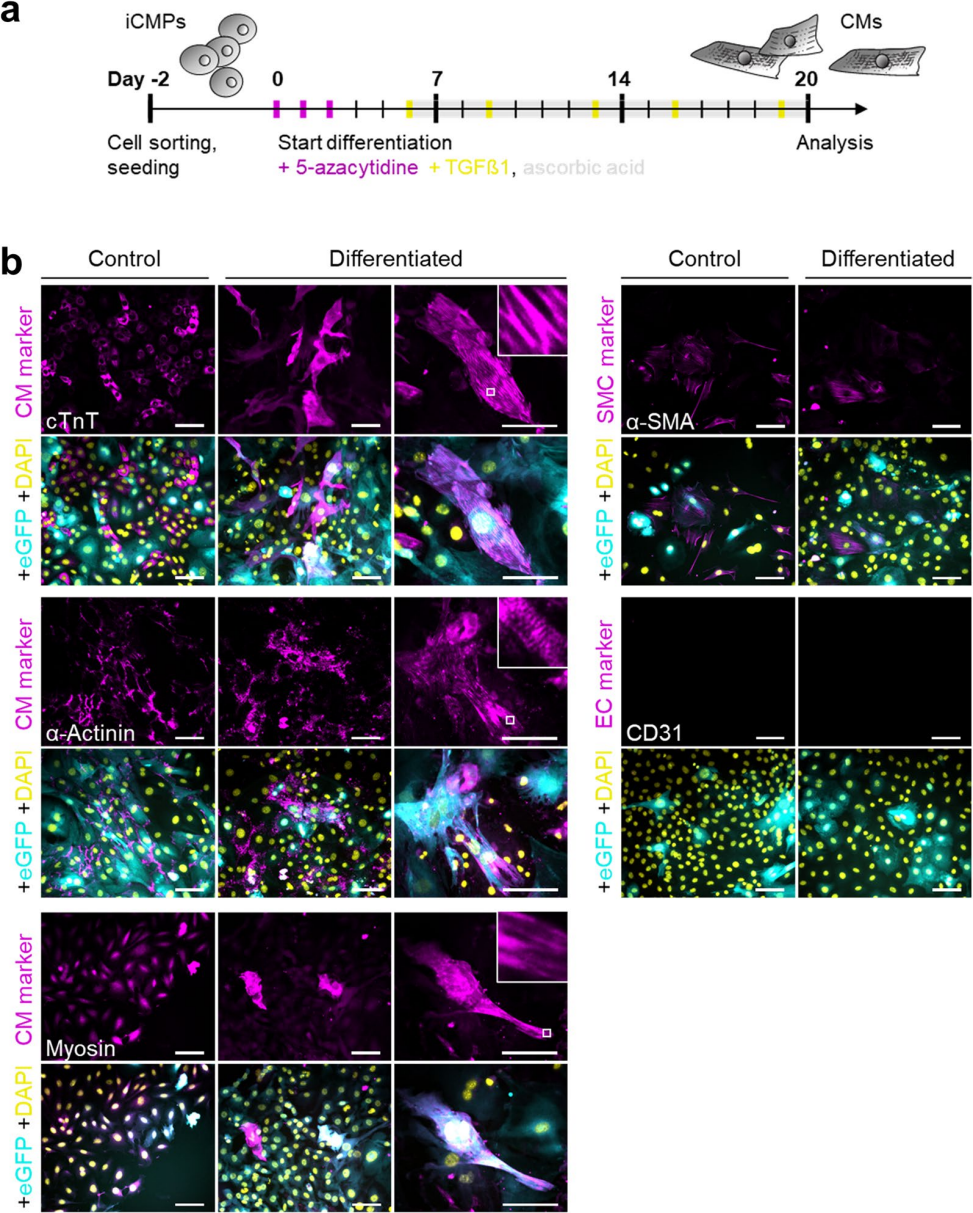
iCMPs differentiate into sarcomere-forming CM-like cells. **a** Schematic outline of the protocol used to differentiate iCMPs into CMs. **b** Immunofluorescent staining for cardiovascular lineage markers (magenta) in combination with eGFP (cyan) in iCMPs 20 days after differentiation initiation. iCMPs differentiate into CM as indicated by sarcomere-like striations in cells labeled by cTnT, α-actinin, and myosin. Neither CD31-positive cells nor an increase in number or fluorescence intensity of alpha smooth muscle actin (α-SMA)-positive cells are evident. DAPI (yellow) marks cell nuclei. Scale bars, 100 µm Figure 5

Having shown that iCMPs display progenitor/precursor features, such as CPC marker expression and self-renewing proliferation capacity, we next explored the ability of iCMPs to give rise to functional CMs, a fundamental requirement for cell products meant to regenerate muscle of infarcted hearts. To initiate differentiation in iCMPs, we followed an established protocol that uses 5-azacytidine and TGFB1 (Fig. 4a) [35]. Within 20 days of differentiation, iCMPs changed their morphology from small size and round shape to large size and rod-like shape. Immunocytological analyses revealed dense, striated expression patterns for the contractile proteins cTnT, α-actinin, and myosin, indicating sarcomere formation (Fig. 4b). However, despite sarcomere assembly, iCMP-derived CMs did not contract spontaneously. Notably, there were no signs for endothelial or smooth muscle cell differentiation as CD31-positive cells were not detected and the number as well as the fluorescence intensity of alpha smooth muscle actin-positive cells did not change (Fig. 4b). There was also no sign for iCMP dedifferentiation. These results indicate that iCMPs are CM precursor-like cells that are able to differentiate into rod-shaped CM-like cells with organized sarcomeres.

### Intramyocardial iCMP transplantation after acute ischemic injury prevents CM loss, attenuates fibrotic scarring, and preserves left ventricular function

**Fig. 5:**
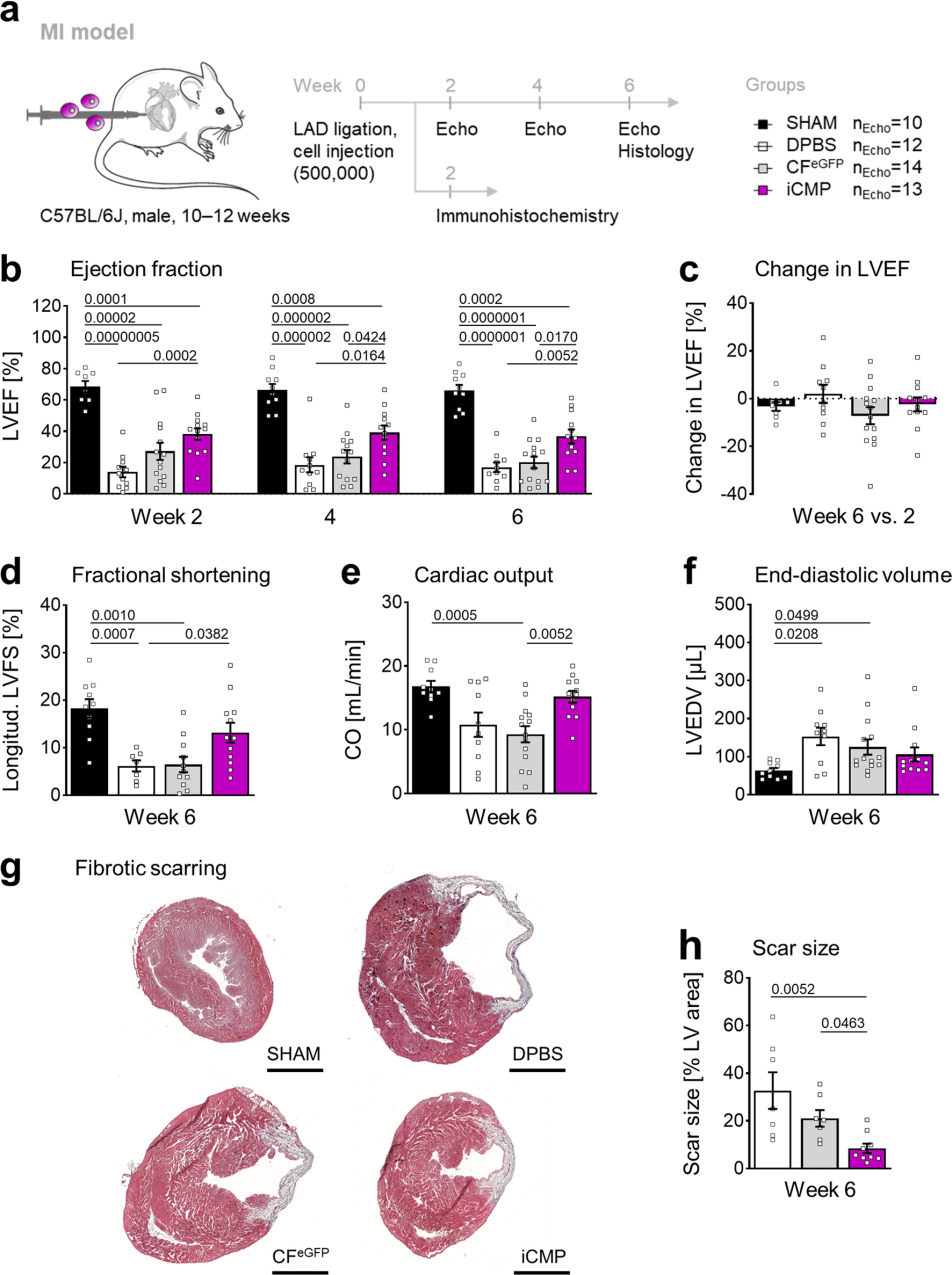
iCMP transplantation attenuates fibrotic scarring and preserves cardiac function after myocardial infarction. **a** Experimental strategy used to evaluate the effect of intramyocardial iCMP transplantation in mice after left anterior descending artery (LAD) ligation and myocardial infarction (MI). Left ventricular performance and geometry were repeatedly measured by transthoracic echocardiography (echo; n_SHAM_ = 10, n_DPBS_ = 12, n_CFeGFP_ = 14, n_iCMP_ = 13 mice). Scar size was determined by histology (n_DPBS_ = 7, n_CFeGFP_ = 7, n_iCMP_ = 9 mice). Cell retention was investigated by immunohistochemistry (n_DPBS_ = 5, n_CFeGFP_ = 5, n_iCMP_ = 6). **b**, **c** Left ventricular pump function is given as LVEF (**b**) or change in LVEF for individual animals over 4 weeks **c**. After iCMP injection, LVEF is stably preserved over the entire follow-up period. **d** Left ventricular contractility is given as longitudinal left ventricular fractional shortening (LVFS). iCMP and SHAM LVFS are not significantly different. **e** Cardiac output (CO), a measure of ejected blood volume, is higher in iCMP animals compared to CF^eGFP^ animals. **f** Left ventricular geometry as determined by left ventricular end-diastolic volume (LVEDV). Compared to SHAM ventricles, DPBS and CF^eGFP^ ventricles are significantly dilated, whereas iCMP ventricle volumes are not significantly different. **g** Representative images of Masson-trichrome–stained heart sections. Red marks muscle fibers, blue marks collagen fibers, light red marks cytoplasm, and dark brown marks cell nuclei. Scale bars, 2 mm. **h** Quantification of scar size in **g** indicates that iCMP injection reduces fibrotic scarring. Scatter plot with bar graph shows mean ± SEM. Overlayed single dots represent individual animals. Cardiac function data were analyzed using a mixed-effects model and Holm-Sidak's post-hoc tests. Change in LVEF data were analyzed using an ordinary one-way ANOVA (F(3, 40) = 1.3, *P* = 0.2755). Additional file 1: Fig. S7b provides additional echocardiography data and statistical test results for all follow-up examinations. Scar size data were analyzed using Kruskal–Wallis test and Dunn’s post-hoc tests (Kruskal–Wallis statistic = 11.18, P_overall_ = 0.0037, P_DPBSvsCFeGFP_ = 1). *P*-values for significant results are shown Figure 6

Having demonstrated that iCMPs are capable of partial CM differentiation in vitro, we next evaluated their therapeutic efficacy in vivo in a murine model of myocardial infarction. For that purpose, we permanently ligated the left anterior descending artery of adult C57BL/6 J hearts, injected 500,000 purified iCMPs into the infarct border zone, and measured cardiac function by transthoracic echocardiography every 2 weeks during a 6-week follow-up (Fig. 5a).

Regarding survival rates, we observed no adverse effect iCMP transplantation, supporting the safety of our cell product (Additional file 1: Fig. S7a). Regarding cardiac performance, the therapeutic effect of iCMPs was already detectable after 2 weeks, with left ventricular ejection fraction (LVEF) of iCMP-treated animals being significantly higher compared to LVEF of DPBS-treated control animals (mean LVEF ± SEM, iCMP: 38.2 ± 3.7%, CF^eGFP^: 27.3 ± 5.5%, DPBS: 14.0 ± 3.3%, SHAM: 68.5 ± 3.6%; Fig. 5b). After 4 and 6 weeks, LVEF in the iCMP group was significantly better preserved compared to LVEF in both myocardial infarction controls, being almost twice as high as in the DPBS and the CF^eGFP^ group (LVEF week 6, iCMP: 36.8 ± 4.4%, CF^eGFP^: 20.2 ± 3.7%, DPBS: 17.0 ± 3.1%; Fig. 5b), but only half as high as in the healthy SHAM control group (66.0 ± 3.7%). Over the 6-week observation period, mean LVEF was stable in the iCMP group, as was mean LVEF change determined for individual animals (Fig. 5c). After 6 weeks, cardiac contractility measured as longitudinal left ventricular fractional shortening was similar in iCMP and SHAM animals and tended to be higher in iCMP compared to CF^eGFP^ and DPBS animals (Fig. 5d). Correspondingly, iCMP animals showed a noticeably higher volume of ejected blood per unit time (cardiac output; Fig. 5e) or per heart beat (stroke volume; Additional file 1: Fig. S7b). Interestingly, left ventricular dilatation, a precursor of left ventricular dysfunction, was attenuated in iCMP-treated animals (Fig. 5f). Both DPBS- and CF^eGFP^-treated animals showed strongly increased left ventricular end-diastolic volumes, whereas iCMP-treated animals showed no statistically significant difference compared to SHAM animals. Analysis of infarct expansion in Masson-trichrome–stained heart sections revealed that the infarct size in the left ventricular free wall was much smaller in iCMP animals after 6 weeks, which was accompanied by higher left ventricular muscle content and reduced left ventricular wall thinning (Fig. 5g, h). Mean scar size was 8.5 ± 2.0% (mean ± SEM) of the left ventricular cross-sectional area in iCMP animals, compared to 21.1 ± 3.5% and 32.7 ± 7.7% in CF^eGFP^ and DPBS animals, respectively.

Knowing that iCMP transplantation after myocardial infarction results in beneficial effects on cardiac structure and function, we finally sought to determine whether iCMPs engrafted into the infarcted myocardium and if so, how long they survived and whether they differentiated into CMs. Because the reprogramming transgenes were coupled to an eGFP reporter gene, we performed immunohistology for eGFP to locate transplanted iCMPs, in combination with cTnI and CD31 to distinguish CMs and endothelial cells from other nonmyocytes. Cross sections were screened for eGFP-based cell retention from the apex to the ligation site using standard fluorescence as well as confocal microscopy. Unexpectedly, despite increased left ventricular muscle content in iCMP-injected hearts, we did not find definite evidence for iCMP retention and engraftment into myocardium based on eGFP expression, in both week-2 and week-6 cross, strongly suggesting eGFP signal and/or cell loss within 2 weeks sections (no 2-day or 1-week group available). Taken together, these results demonstrate that intramyocardial iCMP transplantation in a murine model of acute myocardial infarction preserves left ventricular function and attenuates adverse structural left ventricular remodeling through an as of yet unknown mechanism.

### iCMPs secrete cardioprotective factors

To understand the mechanism behind the therapeutic effect of iCMP transplantation, we analyzed the paracrine activity of iCMPs in comparison to CFs at the mRNA as well as the protein level [37].

For mRNA-based paracrine profiling, we used existing RNA sequencing data and searched for previously documented paracrine factors related to the following cardiac repair processes (comprehensively reviewed in [38]): (1) stimulation of neovascularization to improve tissue blood supply (e.g., *Vegf*, *Pdgf*, *Ang*, *Pigf*), (2) attenuation of apoptosis to limit CM death (e.g., *Hgf*, *Igf*, *Fgf2*, *Stc1*, *Sdf1*), (3) attenuation of fibrosis to prevent adverse ventricular remodeling (e.g., *Timp1*, *Timp2*, *Thbs1*, *Sfrp2*), and (4) attenuation of inflammation to prevent an excessive and or/ chronic inflammatory response (e.g., *Il10*, *Tsg6*, *Ptx3*, *Tnfrsf1a*). Gene expression analysis revealed distinct factor expression profiles with little overlap for CFs and iCMPs (Additional file 1: Fig. S8). Factors with high expression levels in iCMPs had low expression levels in CFs and vice versa. It was also striking that in iCMP samples, the expression levels for the majority of factors were higher, including those for proinflammatory factors.

**Fig. 6:**
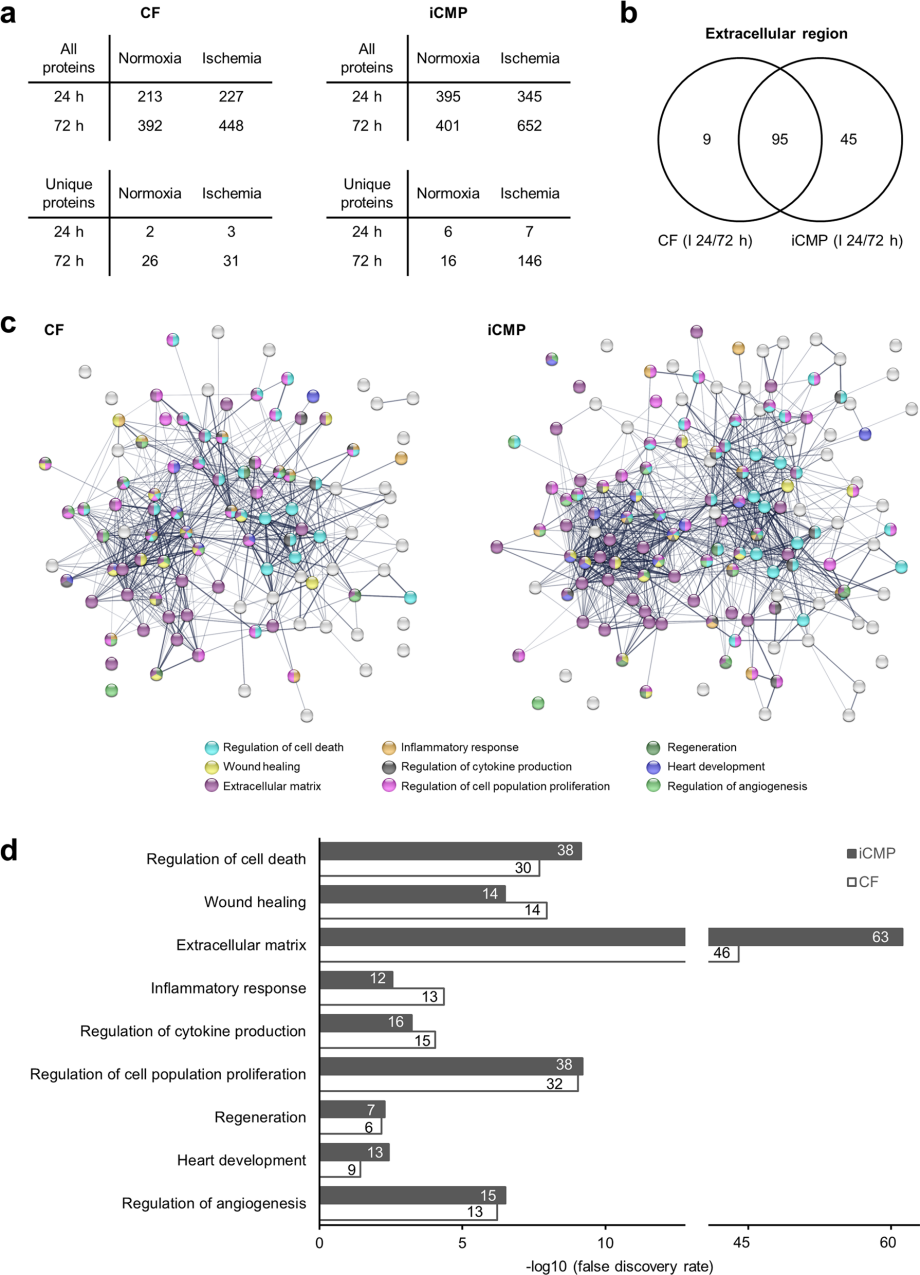
Proteomic analysis of iCMP and CF secretomes by mass spectrometry reveals distinct quantitative and qualitative paracrine expression profiles **a** Number of total and unique proteins detected by mass spectrometry in medium supernatants of iCMPs (*n* = 3) and CFs (*n* = 3) cultured under normoxic (21% O_2_, 1 g/l glucose) or ischemic (1% O_2_, no glucose) conditions for 24 h or 72 h, revealing a superior secretory activity for iCMPs under ischemic conditions. **b** Venn diagram for extracellular proteins (gene ontology term 0005576 “extracellular region”) of CFs (104 proteins) and iCMPs (140 proteins) cultured under ischemia **I** for 24–72 h. Single proteins are listed in Additional file 1: Table S3. **c** Protein interaction networks and functional enrichment analysis for biological processes and cellular components related to cardiac repair for proteins of CFs and iCMPs in b, generated using the STRING database. **c** Bar graph showing the enriched biological processes and cellular components related to cardiac repair for iCMPs and CFs, the annotated protein count, and the significance of enrichment. The iCMP secretome is significantly enriched with proteins associated with the regulation of cell death, extracellular matrix, and heart development

For the protein-based paracrine profiling, we performed mass spectrometric analysis on media supernatans from iCMP and CFs cultured for 24 h or 72 h under infarct-like, ischemic conditions. Here, we also found distinct protein secretion profiles for each cell type. The number of all detected proteins alone was significantly higher in iCMP samples compared to CF samples, both after 24 h (number of detected proteins: iCMPs vs CFs, 345 vs 227) and after 72 h of ischemia (652 vs 448 proteins) (Fig. 6a, upper tables). Similar numbers of proteins were detected in media supernatants of CFs after 72 h ischemia or normoxia (448 vs 392 proteins), whereas significantly more proteins were found in media supernatants of iCMPs after 72 h ischemia compared to normoxia (652 vs 401 proteins) (Fig. 6a, upper tables). This phenomenon was also evident for proteins unique to each condition (Fig. 6a, lower tables).

We then focused on extracellular proteins secreted by iCMPs and CFs under ischemia (24 and 72 h) (Fig. 6b) and subjected these proteins to network and functional enrichment analyses for cardiac repair processes (Fig. 6c). We found that the iCMP secretome not only contained more proteins involved in the regulation of cell death, extracellular matrix composition, or heart development, but also that the significance of enrichment for these processes was much higher (Fig. 6d). By contrast, for the CF secretome, the significance of enrichment for biological processes such as wound healing (including negative regulation of wound healing), inflammatory response (including heart inflammation), and cytokine production was higher, despite comparable annotated protein counts.

Among the proteins secreted exclusively by iCMPs, we noticed galectin-3 (LGALS3; Additional file 1: Table S3) and the protein S100–A10 (S100A10). Galectin-3 has been reported to have beneficial effects on the outcome of wound healing, infarct size, macrophage infiltration, fibrosis, ventricular remodeling, ventricle dilation, and heart function, particularly when present in the early phase after myocardial infarction [39]. The protein S100-A10 (S100A10) is reportedly associated with macrophage invasion and migration via MMP9 signaling, although as of yet in the context of tumor growth and metastasis (reviewed in [40]). In summary, these results suggest that iCMPs display a quantitative superior paracrine activity compared to CFs and that the qualitative secretome composition differs in terms of factors related to CM survival, wound healing, extracellular matrix deposition, inflammatory response, cytokine production and heart development.

## Discussion

In the present study, we provide evidence that enforced *GMTMy* expression converts heart-derived fibroblasts into expandable cells with a pre-cardiomyocyte phenotype that can be purified based on *Myh6/7* expression using molecular beacon technology. After myocardial infarction, intramyocardial transplantation of these induced precursors (iCMPs) resulted in decreased scar size and preserved cardiac contractility. To the best of our knowledge, this is the first report on therapeutically active, CM precursor-like cells generated by direct fibroblast reprogramming and molecular beacon selection.

GMTMy activated global cardiac gene regulatory networks and induced the expression of a broad spectrum of CM lineage markers in fibroblasts, with our induction efficiencies being in the range of previously published direct cardiac reprogramming protocols [13]. Surprisingly, GMTMy also preserved mitotic activity and induced CPC markers, pointing toward the generation of proliferating iCM precursors in parallel to quiescent iCMs. Previous studies found no signs for the simultaneous emergence of CMs and their precursors [14, 41–43], although this scenario is not far-fetched. Cellular reprogramming usually proceeds asynchronously, resulting in heterogenous cell populations with various intermediate conversion stages [14, 44–46]. Possible explanations for our observation may include: (1) iCMPs emerge specifically after *GMTMy* transduction, (2) iCMP generation and maintenance is supported by regular subculture that impedes iCM formation, and (3) other protocols induce only a transient iCMP state that is easily overlooked. It remains to be determined whether the iCMP state overlaps with normal CM development or whether it is completely artificial. Single-cell-based transcriptome analyses will also be of interest to assess priming for specific CM subtypes. With regard to clinical translation, the use of nonintegrating Sendai viruses instead of lentiviruses or a chemical equivalent for the transgene-driven lineage conversion protocol is desirable, as certainly is the testing of adult and noncardiac fibroblast sources.

Obtaining a pure cell population after reprogramming is imperative for any future therapeutic application. We selected iCMPs based on intracellular *Myh6/7* mRNA expression and enriched them by fluorescence-activated cell sorting. To avoid the use of a transgenic reporter system, which would complicate both experimental protocols and clinical translation, we used molecular beacon technology, a genetic-modification–free platform that uses transfectable mRNA hybridization probes for cell labeling and is compatible with clinical use. The *Myh6/7* beacons used in this study have previously been used to purify human induced pluripotent stem cell(iPSC)-derived cells, which integrated into ischemic myocardium and elicited therapeutic benefits after transplantation in a mouse model of myocardial infarction [47]. Our in vivo results are consistent with these observations and demonstrate the suitability of the molecular beacon technology to generate therapeutic cell products. Due to the sensitivity of molecular beacons to enzymatic degradation, their intracellular presence is of short duration, minimizing side effects after cell transplantation. Importantly, the molecular beacon portfolio could easily be extended to subpopulations of ventricular-, atrial-, and/or pacemaker-primed iCMPs, as demonstrated for the purification of IRX4(iroquois homeobox 4)–positive ventricular CMs from differentiating embryonic stem cells [48]. Interestingly, human iPSC-derived CM-fated progenitors have been sorted based on the cell surface protein CD82 [49], and our RNA sequencing data show higher CD82 expression in iCMPs compared to cardiac fibroblasts and adult heart tissue. However, because CD82 is implicated in cell proliferation rather than CM fate decision, CD82 alone may be insufficient to enrich iCMPs and additional CM-specific markers would be required.

To demonstrate a genuine precursor character of iCMPs, we evaluated long-term self-renewal and cardiac differentiation. Regarding phenotype maintenance during expansion, current protocols for tripotent iCPCs and iPSC-derived CPCs require either complex factor cocktails [20, 21, 50, 51] or genetic modification [52]. By contrast, our ascorbic acid approach for unipotent CM precursors is surprisingly simple. A hypothetic explanation for this discrepancy may be that the complex phenomenon of tripotency requires more advanced maintenance efforts than unipotency does. Regarding the mechanistic role of ascorbic acid, we assume that its continued presence is essential not only to prevent iCMPs from reverting back to a fibroblast phenotype but also to sustain proliferation capacity. Our findings are corroborated by previous studies showing that ascorbic acid can indeed maintain the proliferation and differentiation potential of numerous stem and progenitor cells by preventing senescence and supporting epigenetic plasticity (reviewed in [53]). Moreover, in the context of CM generation, ascorbic acid has been shown to enhance CPC proliferation and to promote the cardiomyogenesis of pluripotent stem cells via modulation of SMAD-signaling and synthesis of extra cellular matrix proteins [54, 55], which is why ascorbic acid has now evolved to a standard supplement for routine CM production. Expansion of iCMPs for 27 days generated ~ 6 × 10^6^ iCMPs for every plated cell, with an average population doubling time of 29.3 ± 4.9 h, and no signs for decreasing proliferation capacity during 4 weeks of observation. Thus, iCMP expansion characteristics are similar to those previously reported for tripotent iCPCs [20]. With regard to clinical use of iCMP technology, the fast proliferation kinetics will facilitate the production of therapeutic doses in the magnitude of one billion cells, probably even for repeated administrations [56]. Nevertheless, further research is warranted regarding the performance of adult, extra-cardiac, or patient-derived cells, where age- and disease-related impairment of proliferation may become relevant.

Regarding differentiation, exposure of iCMPs to an established signaling cocktail containing 5-azacytidine and TGFB1 unlocked iCMP phenotype, initiated terminal CM differentiation, and resulted in the formation of sarcomere-forming cells. In our hands iCMPs did not beat spontaneously. We assume that iCMP-derived CMs require additional soluble, metabolic, topographic, electrical, mechanical, and/or cellular cues for maturation provided either by cardio-mimetic in vitro systems or the native cardiac in vivo environment. Polyinosinic‐polycytidylic acid-based epigenetic priming of progenitor cells may be another option to enhance CM maturation [57].

In a mouse model of myocardial infarction, intramyocardial iCMP transplantation resulted in smaller scar size and higher LVEF compared to DPBS injection or CF^eGFP^ transplantation. This therapeutic effect of unipotent iCMPs is in agreement with previous observations in direct reprogramming studies made after tripotent iCPC transplantation or in vivo reprogramming of infarct fibroblasts into iCMs in rodent models of myocardial infarction (Table 1) [13]. Therapeutic effect occurrence and sustainability were also similar; group differences of cardiac parameters were first detected after 2–4 weeks and sustained for 4–12 weeks in longitudinal studies. Interestingly, the comparison of mean group differences (treatment – placebo) for LVEF and scar size across numerous studies suggests that iCPC transplantation is more effective in preserving LVEF, whereas in vivo reprogramming is more effective in preventing fibrotic scarring, and that iCMP transplantation is highly effective in both preserving function and preventing fibrosis (Table 1). Moreover, the comparison of outcomes after transplantation of artificially induced progenitors/precursors derived from young and healthy cell sources and their naturally occurring endogenous counterparts derived from adult heart biopsies suggests a superior therapeutic potential for iCPCs and iCMPs (Table 1) [13, 58]. Age and disease may be responsible for this phenomenon and have to be taken into consideration when it comes to choosing a starting cell source for iCMP generation during clinical development.

**Table 1:** Comparative outcome analysis for different therapeutic approaches in rodent models of acute myocardial infarction

Therapeutic approach	Outcome heart function: LVEF		Outcome tissue remodeling: scar size		References
	Mean group difference^a^ (MIN, MAX) [%]	Study number	Mean group difference^a^ (MIN, MAX) [%]	Study number	
iCMP transplantation after ex vivo fibroblast reprogramming	20	1	− 23	1	This study
iCPC transplantation after ex vivo fibroblast reprogramming	20 (6, 30)	3	− 13 (− 5, − 20)	2	Reviewed in [13]
Endogenous CPC transplantation after ex vivo expansion	12 (0.3, 26)	70	− 8 (− 0.8, − 34)	23	Reviewed in [58]
In vivo reprogramming of infarct fibroblasts into iCMs	11 (4, 27)	7	− 19 (− 5, − 40)	7	Reviewed in [13]

^a^Group difference = treatment – placebo control

The mechanism of action behind the therapeutic effect of iCMP transplantation can, in the absence of cell retention evidence, not be attributed to direct but only to indirect, paracrine effects. We assume that the harsh conditions in the infarcted heart affected cell retention and that the strong GFP autofluorescence of cardiac tissue interfered with reliable cell detection. Cell injection into healthy SHAM animals and the use of modified reporter systems are possible future experimental scenarios to test these assumptions.

To evaluate the paracrine effect of iCMPs and CFs on infarcted hearts, we performed secretory profiling at the mRNA and protein level. Our results suggest that compared to the CF secretome, the iCMP secretome is more complex, containing more proteins, and that many of the proteins are related to a balanced, well-orchestrated healing process after myocardial infarction that limits infarct size, prevents remote fibrosis, counteracts adverse remodeling, limits ventricle dilation, and preserves cardiac function through the secretion of pro-survival factors that prevent CMs death, an acute inflammatory impulse, the infiltration of macrophages and their phagocytotic activity to clear dead cells, extracellular matrix deposition and scar formation to replace lost muscle tissue and to maintain cardiac integrity.

The differences in the secretory profiles of iCMPs and CFs are probably due to their different geno- and phenotypes. However, the complexity and versatility of the iCMP secretome is surprising and raises the question of whether a cell-free therapy would be useful.

In the present study, we used shotgun mass spectrometry to investigate the secreted proteins that contribute significantly to the in vivo performance of iCMPs. In addition to the identified proteins, small cytokines or miRNAs may also play a crucial role in the secretome and should be investigated in future studies.

### Limitations

The general limitations of such experimental studies are manifold and have been highlighted before [13], and our series of experiments is no exception. The mechanism-of-action is not completely clear. Viable cells could not be detected weeks after transplantation. Instead, we found that iCMPs have a distinct secretory profile, which may explain their beneficial effects. However, identification of the exact mechanism-of-action of a given secreted protein on a given aspect of myocardial biology was beyond the scope of our investigation. Similarly, the question of the “proper” control group cannot be answered conclusively. Is every step of our reprogramming and purification protocol absolutely necessary with regard to improving heart function? Could purification be omitted etc.? A definitive answer cannot be provided. Based on the body of knowledge available at the time, we developed this particular protocol to yield a cell population with certain pre-defined characteristics and hypothesized that it will have beneficial effects on the ischemic mouse myocardium. This we could confirm, but that does not mean that modifications of our protocol or altogether different strategies of cell-based therapies cannot be equally or even better suited. In recent years it became abundantly clear that regenerating the myocardium via cell therapy is much more complex than initially hoped for. In this regard, the validity of our experimental approach is not different from many others, and a vast amount of research work remains to be done in order to identify the “best” approach, if there is one.

## Conclusion

GMTMy directly reprograms cardiac fibroblasts into proliferative iCMPs that can be selected for *Myh6/7* expression using molecular beacon technology and expanded to generate therapeutically relevant cell doses. Given their positive effects in the myocardial infarction model, further studies are needed to elucidate the detailed mechanism-of-action and to assess the potential for future translational development.

### Supplementary Information

**Additional file 1 Fig. S1: **Lentiviruses enable efficient transgene delivery into primary cardiac fibroblasts. **Fig. S2:** Preliminary experiments for reprogramming factor selection. **Fig. S3:** GMTMy induces CM-specific protein expression. **Fig. S4:** Gating strategy used for flow cytometric analysis of cTnT protein expression. **Fig. S5:** Myh6/7 molecular beacons can be efficiently delivered into adherent cells and specifically label CMs. **Fig. S6:** Gene expression analyses using RNA sequencing and reverse transcription-quantitative PCR reveal similar expression patterns. **Fig. S7:** iCMP transplantation after myocardial infarction preserves left ventricular performance. **Fig. S8: **Explorative analysis of global transcriptome data for paracrine factors implicated in cardiac repair suggests a cardioprotective secretome for iCMPs. **Table S1:** Antibody details. **Table S2:** Reverse transcription-quantitative PCR primer sequences. **Table S3** Proteins of the secretome (gene ontology term 0005576 “extracellular region”) of iCMPs and CFs as detected by mass spectrometry and their assignment to biological processes and cellular components related to cardiac repair.

## Data Availability

The datasets supporting the conclusions of this article are included within the article and its additional file. RNA sequencing data sets are publicly available via the NCBI Gene Expression Omnibus; the accession number is GSE159315 (https://www.ncbi.nlm.nih.gov/geo/query/acc.cgi?acc=GSE159315). The mass spectrometry proteomics data have been deposited to the ProteomeXchange Consortium [59] via the PRIDE [60] partner repository with the dataset identifier PXD042806 (https://proteomecentral.proteomexchange.org/cgi/GetDataset?ID=PXD042806).

## Abbreviations

BHQ2: Black Hole Quencher 2
CF^eGFP^: *eGFP*-transduced cardiac fibroblast
CM: Cardiomyocyte
CPC: Cardiac progenitor cell
cTnT: Cardiac muscle troponin T
Cy5: Cyanine 5
DPBS: Dulbecco's phosphate-buffered saline
eGFP: Enhanced green fluorescent protein
GMTMy: GATA4, MEF2C, TBX5, and MYOCD
iCM: Induced cardiomyocyte
iCMP: Induced cardiomyocyte precursor
iCPC: Induced cardiac progenitor cell
iPSC: Induced pluripotent stem cell
LVEF: Left ventricular ejection fraction
PDL: Population doubling level
