# Supplementary material for: Cardiomyocyte precursors generated by direct reprogramming and molecular beacon selection attenuate ventricular remodeling after experimental myocardial infarction
Source: Stem Cell Res Ther. 2023 Oct 15;14:296. doi: 10.1186/s13287-023-03519-w (PMC10577947; doi:10.1186/s13287-023-03519-w)
Supplement: Supplementary file 1 — Additional file 1 Fig. S1: Lentiviruses enable efficient transgene delivery into primary cardiac fibroblasts. Fig. S2: Preliminary experiments for reprogramming factor selection. Fig. S3: GMTMy induces CM-specific protein expression. Fig. S4: Gating strategy used for flow cytometric analysis of cTnT protein expression. Fig. S5: Myh6/7 molecular beacons can be efficiently delivered into adherent cells and specifically label CMs. Fig. S6: Gene expression analyses using RNA sequencing and reverse transcription-quantitative PCR reveal similar expression patterns. Fig. S7: iCMP transplantation after myocardial infarction preserves left ventricular performance. Fig. S8: Explorative analysis of global transcriptome data for paracrine factors implicated in cardiac repair suggests a cardioprotective secretome for iCMPs. Table S1: Antibody details. Table S2: Reverse transcription-quantitative PCR primer sequences. Table S3 Proteins of the secretome (gene ontology term 0005576 “extracellular region”) of iCMPs and CFs as detected by mass spectrometry and their assignment to biological processes and cellular components related to cardiac repair. [file 13287_2023_3519_MOESM1_ESM.docx]

# **Supplementary Information**

**Cardiomyocyte precursors generated by direct reprogramming and molecular beacon selection attenuate ventricular remodeling after experimental myocardial infarction**

Dipthi Bachamanda-Somesh, Kristin Klose, Janita A. Maring, Désirée Kunkel, Karsten Jürchott, Stephanie I. Protze, Oliver Klein, Grit Nebrich, Matthias Becker, Ulrike Krüger, Timo Z. Nazari-Shafti, Volkmar Falk, Andreas Kurtz, Manfred Gossen, Christof Stamm

**
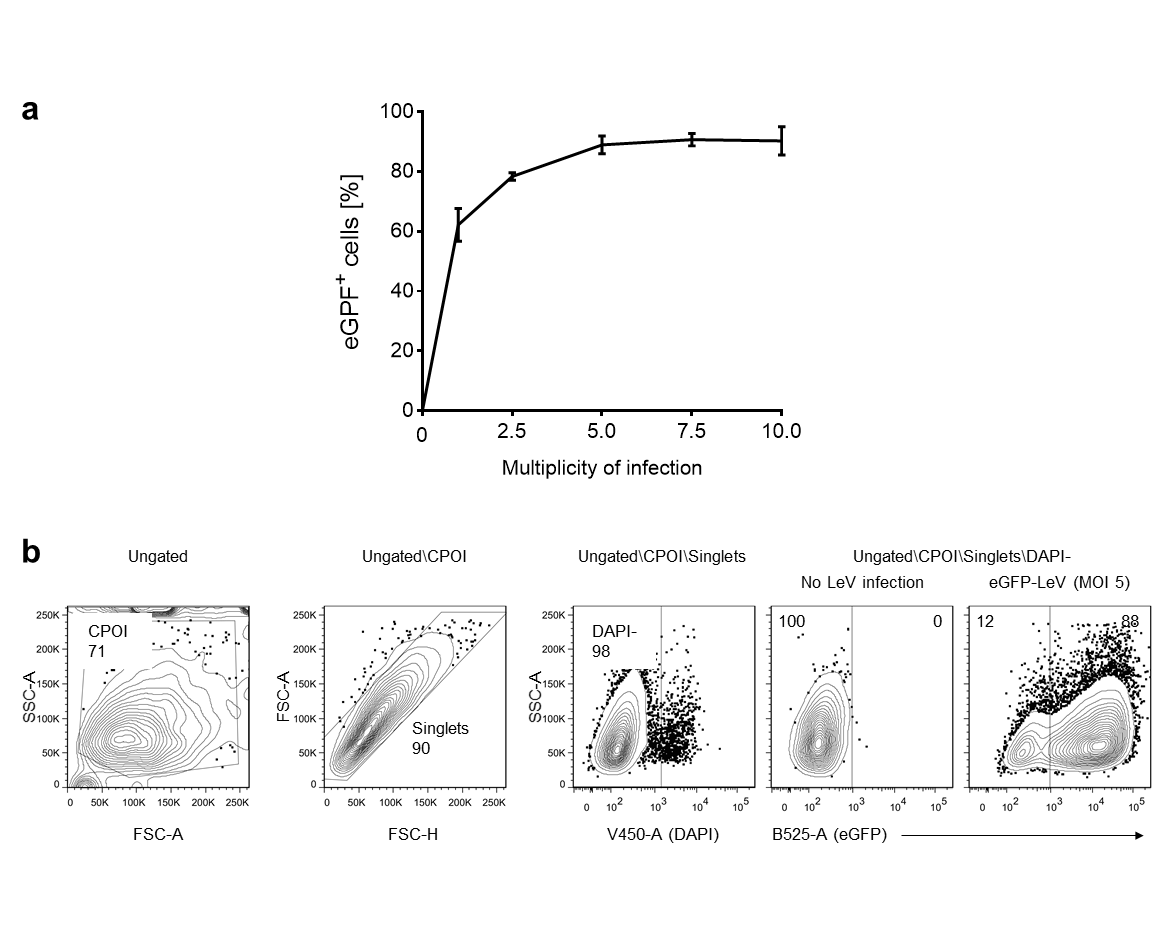
**

## Fig. S1

**Fig. S1** **Lentiviruses enable efficient transgene delivery into primary cardiac fibroblasts**. **a** Assessment of lentiviral transduction efficiency in cardiac fibroblasts by flow cytometry 3 days after eGFP-lentivirus (LeV) infection. At a multiplicity of infection (MOI) of 5, the transduction efficiency was 89 ± 3 %. Increasing the multiplicity of infection beyond 5 did not increase the transduction efficiency, but did increase cell death. Line graph shows mean ± SD (n=2). **b** Contour plots (contour level 5 %) show gating strategy used for flow cytometric analysis of eGFP expression in live, DAPI-negative cells. Quantitative data in all gates are given as percentage of the respective parent population. CPOI, cell population of interest


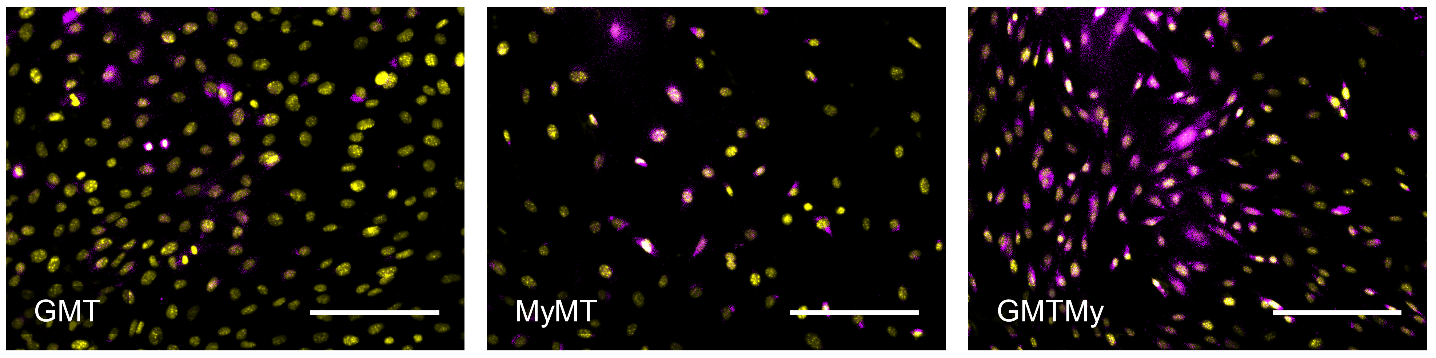


## Fig. S2

**Fig. S2 Preliminary experiments for reprogramming factor selection.** The cardioinductive reprogramming factor combinations GMT (GATA4, MEF2C, TBX5), MyMT (MYOCD, MEF2C, TBX5) and GMTMy (GATA4, MEF2C TBX5, MYOCD) were selected based on a literature search [13]. CF were transduced with these combinations to identify, which combination was most effective in inducing cTnT protein expression. Immunofluorescent staining for cTnT (magenta) 6 weeks post lentiviral infection reveals GMTMy as the most promising candidate (% cTnT+ cells per total cells determined by image analysis: GMT, 8%; MyMT, 24%; GMTMy, 44%). DAPI (yellow) marks cell nuclei. Scale bars, 200 µm


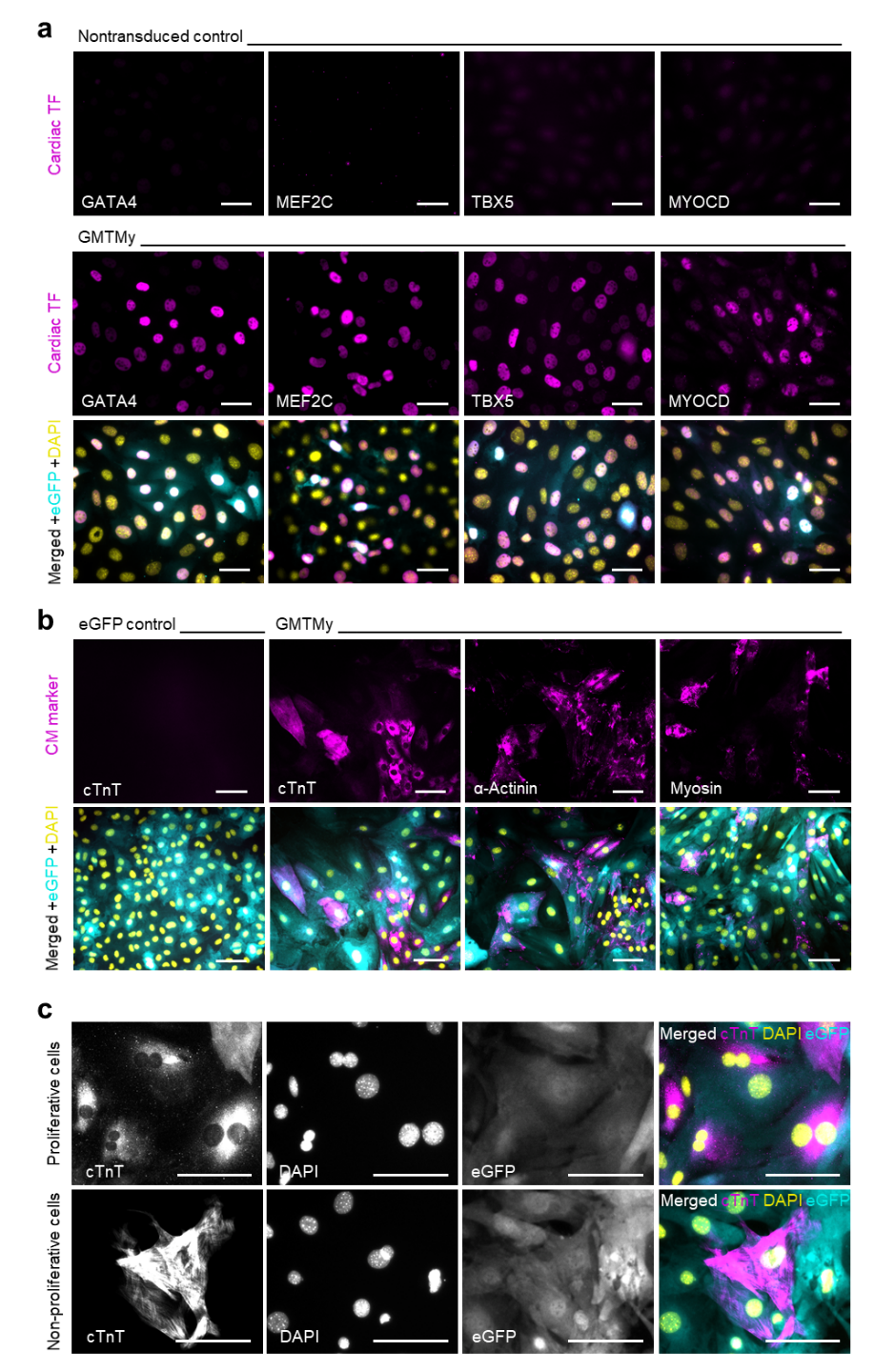


## Fig. S3

**Fig. S3** **GMTMy induces CM-specific protein expression.** **a** Immunofluorescent staining for GATA4, MEF2C, TBX5, or MYOCD (magenta) in nontransduced cardiac fibroblasts (marginal) and in fibroblasts (strong) 3 days after lentivirus infection confirms nuclear overexpression of cardiac transcription factors (TF). Similar overexpression patterns were observed in *GMTMy-*cotransduced cardiac fibroblasts. eGFP (cyan) marks transduced cells, DAPI (yellow) marks cell nuclei. Scale bars, 50 µm. **b** Immunofluorescent staining for cTnT, α-actinin, and myosin (magenta) in combination with eGFP (cyan) 14 days after infection with the GMTMy-lentivirus-cocktail indicates beginning transition toward CM fate. DAPI (yellow) marks cell nuclei. Scale bars, 100 µm. **c** Immunofluorescent staining for CM marker cTnT (magenta) in combination with eGFP (cyan) 14 days after GMTMy-lentivirus infection. DAPI (yellow) marks cell nuclei. Scale bars, 100 µm. Black-and-white images represent single channels, color images represent merged channels of high-magnification immunofluorescence images provided in Fig. 1b, showing that GMTMy coinduced immature, proliferating and more mature, nonproliferative cTnT-positive cells


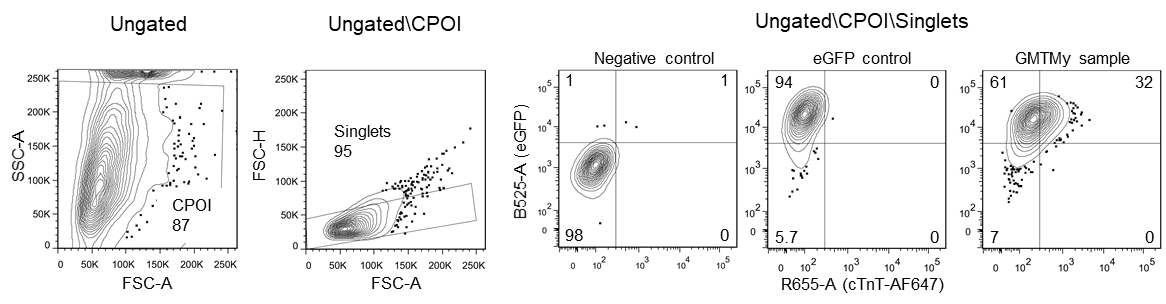


## Fig. S4

**Fig. S4** **Gating strategy used for flow cytometric analysis of cTnT protein expression.** Shown are contour plots (contour level 5 %). Quantitative data in all gates are given as percentage of the respective parent population. CPOI, cell population of interest

**
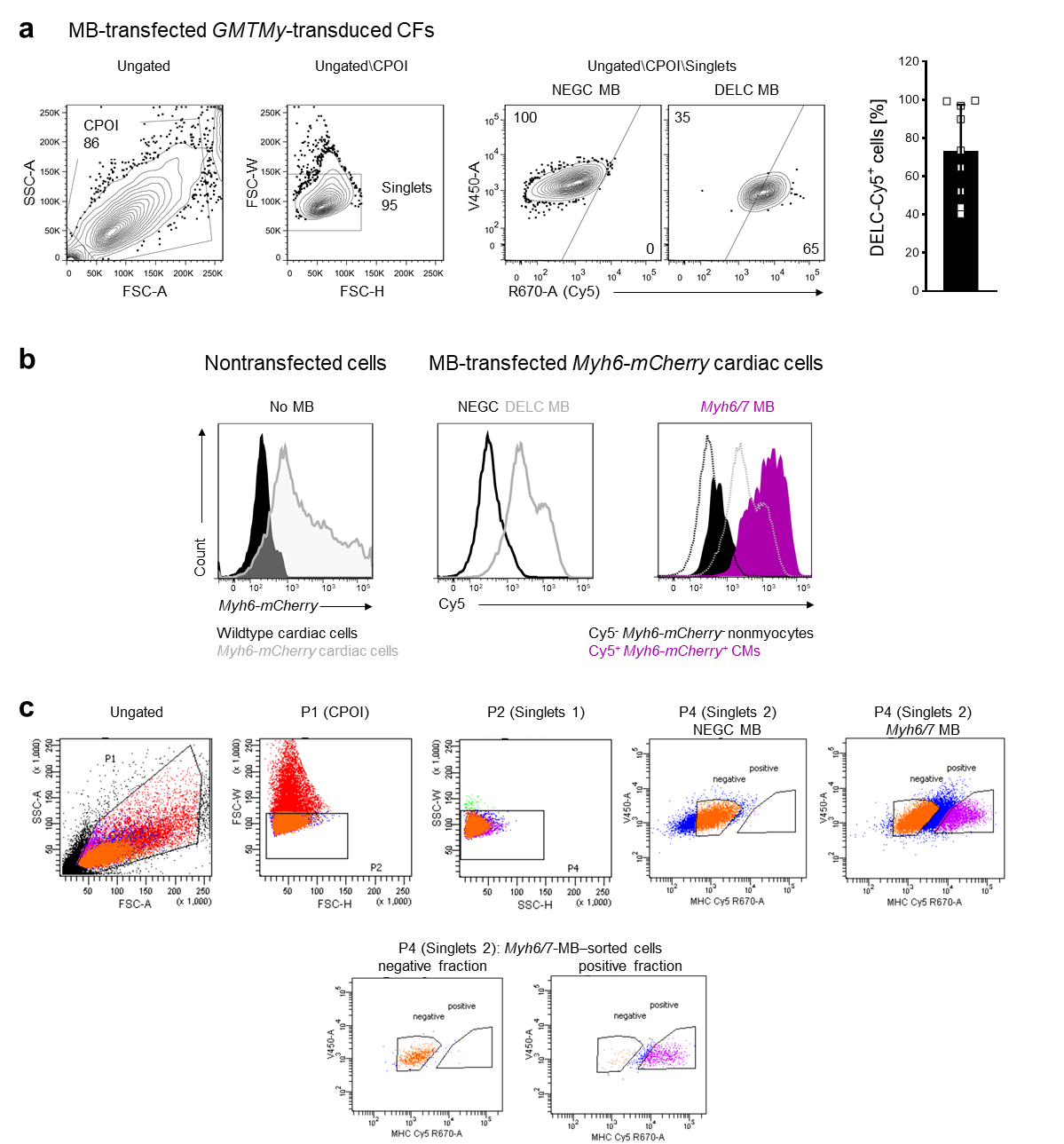
**

## Fig. S5

**Fig. S5** ***Myh6/7* molecular beacons can be efficiently delivered into adherent cells and specifically label CMs.** **a** The *Myh6/7*-beacon–based sorting protocol was established using negative control (NEGC, no sequence match, Cy5-BHQ2 label), delivery control (DELC, nonspecific sequence, Cy5-Cy5 label, no quencher), and *Myh6/7* probes (Cy5-BHQ2 label), which produce Cy5-fluorescence either after probe degradation (negative control), regardless of conformation (delivery control), or after hybridization to *Myh6/7* mRNA (*Myh6/7*). Molecular beacon (MB) transfection efficiency determined by flow cytometry using *GMTMy*-transduced cardiac fibroblasts transfected with negative and delivery control probes amounted to 73.3 ± 24.2 %, respectively. Contour plots (contour level 5 %) show the gating strategy and representative samples. Quantitative data in all gates are given as percentage of the respective parent population. The scatter plot with bar graph presents the proportion of Cy5-positive cells; shown are replicates and mean ± SD (n=9). **b** Assessment of molecular beacon specificity using cardiac cells isolated from wildtype and *Myh6*-*mCherry*–transgenic neonatal mice, transfected with negative control and *Myh6/7* probes and analyzed by flow cytometry. The overlayed histograms show that Cy5 fluorescence overlaps with *mCherry* positivity, demonstrating the specificity of the *Myh6/7* probes for myocytes. **c** Dot plots show gating strategy used for iCMP sorting. P1, population1; CPOI, cell population of interest

**
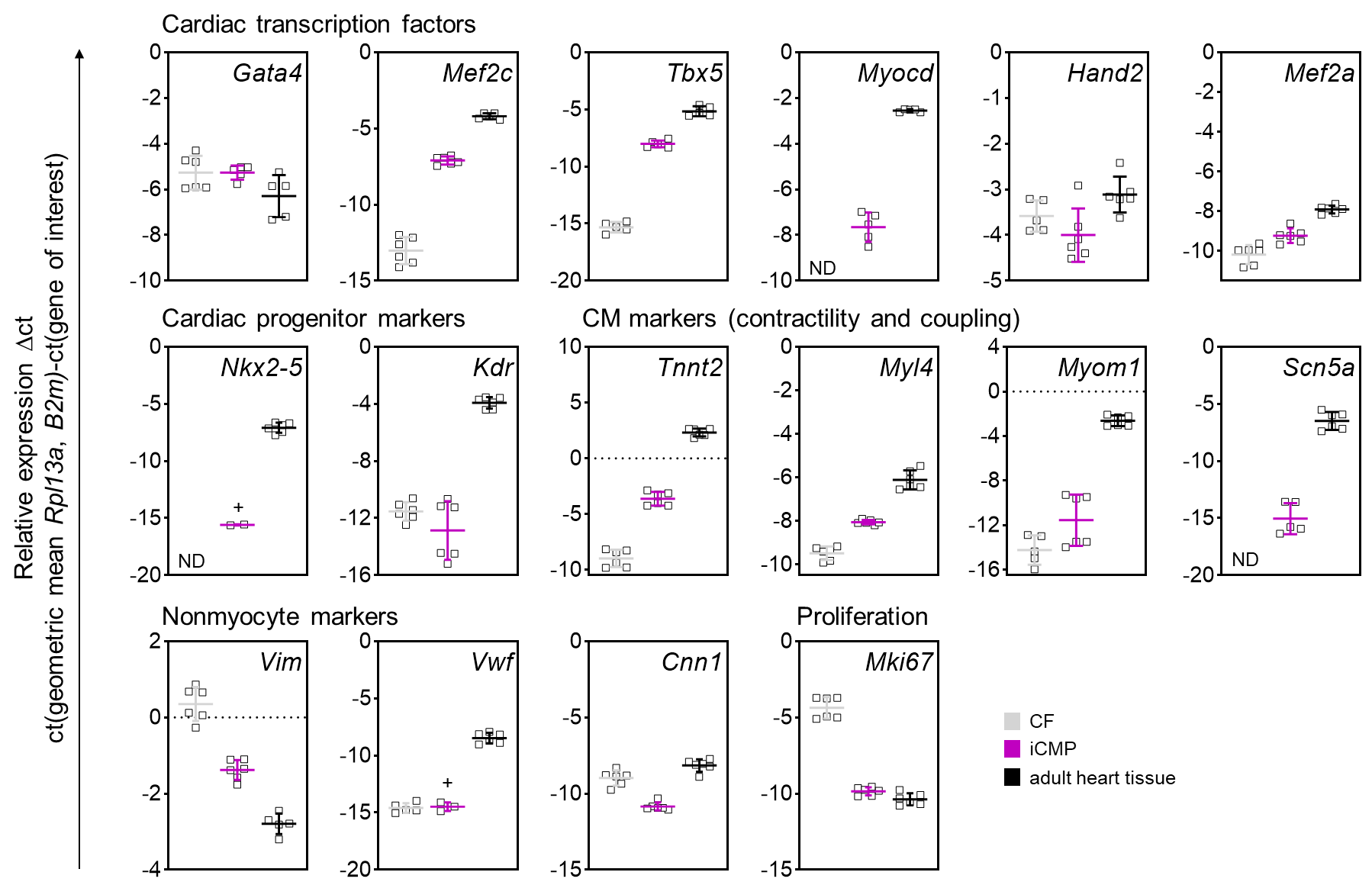
**

## Fig. S6

**Fig. S6** **Gene expression analyses using RNA sequencing and reverse transcription-quantitative PCR reveal similar expression patterns.** Validation of RNA sequencing data for selected genes by reverse transcription-quantitative PCR. Relative gene expression levels of cardiac transcription factors, cardiac progenitor markers, CM markers, nonmyocyte markers, and a proliferation marker are shown for cardiac fibroblasts (CF), purified iCMPs, and adult heart tissue. Gene expression was quantified using the ∆ct-method and was normalized to two references genes (*B2m*, *Rpl13a*). The simultaneous upregulation of progenitor and CM gene expression in iCMPs was confirmed. Nonmyocyte gene expression was not induced in iCMPs compared to fibroblasts. Skeletal muscle marker myogenic differentiation 1 (*Myod1*) was not detectable in any group. Proliferation marker gene expression was markedly reduced but still detectable, supporting the observation of continuous cell proliferation. Scatter plot graphs present replicates and mean ± SD (n=6) (ND, not detected; +, detected in a subset of samples)


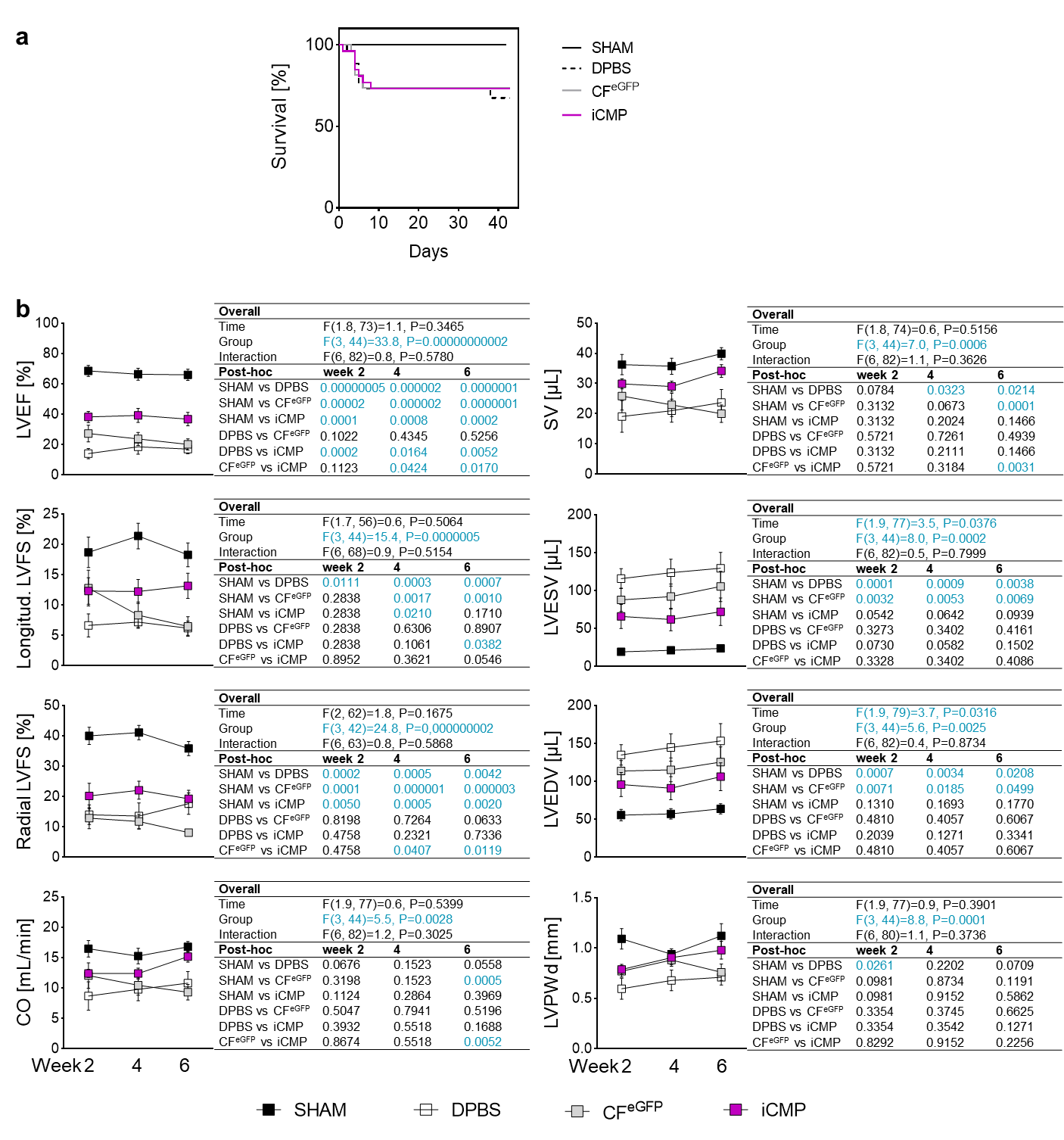


## Fig. S7

**Fig. S7** **iCMP transplantation after myocardial infarction preserves left ventricular performance.** **a** Kaplan-Meier survival analysis (n_SHAM_=10, n_DPBS_=12, n_CFeGFP_=14, n_iCMP_=13 mice). Staircase graph shows that iCMP transplantation is not associated with increased mortality, supporting their safety for therapeutic application. **b** Analysis of cardiac function and geometry by transthoracic echocardiography. Provided are complete sets of longitudinal data and corresponding statistical analysis results for in vivo experiments outlined in Fig. 5. Cardiac pump function is given as LVEF. Heart contractility is given as longitudinal and radial left ventricular fractional shortening (LVFS). Blood volume supplied to the body is given as cardiac output (CO) and stroke volume (SV). Ventricle geometry is given as left ventricular end-systolic volume (LVESV), left ventricular end-diastolic volume (LVEDV), and left ventricular posterior wall thickness at end diastole (LVPWd). Line graphs show mean ± SEM (n_SHAM_=10, n_DPBS_=12, n_CFeGFP_=14, n_iCMP_=13 mice). Data were analyzed using a mixed-effects model and Holm-Sidak's post-hoc tests. Significant results with P<0.05 are shown in cyan


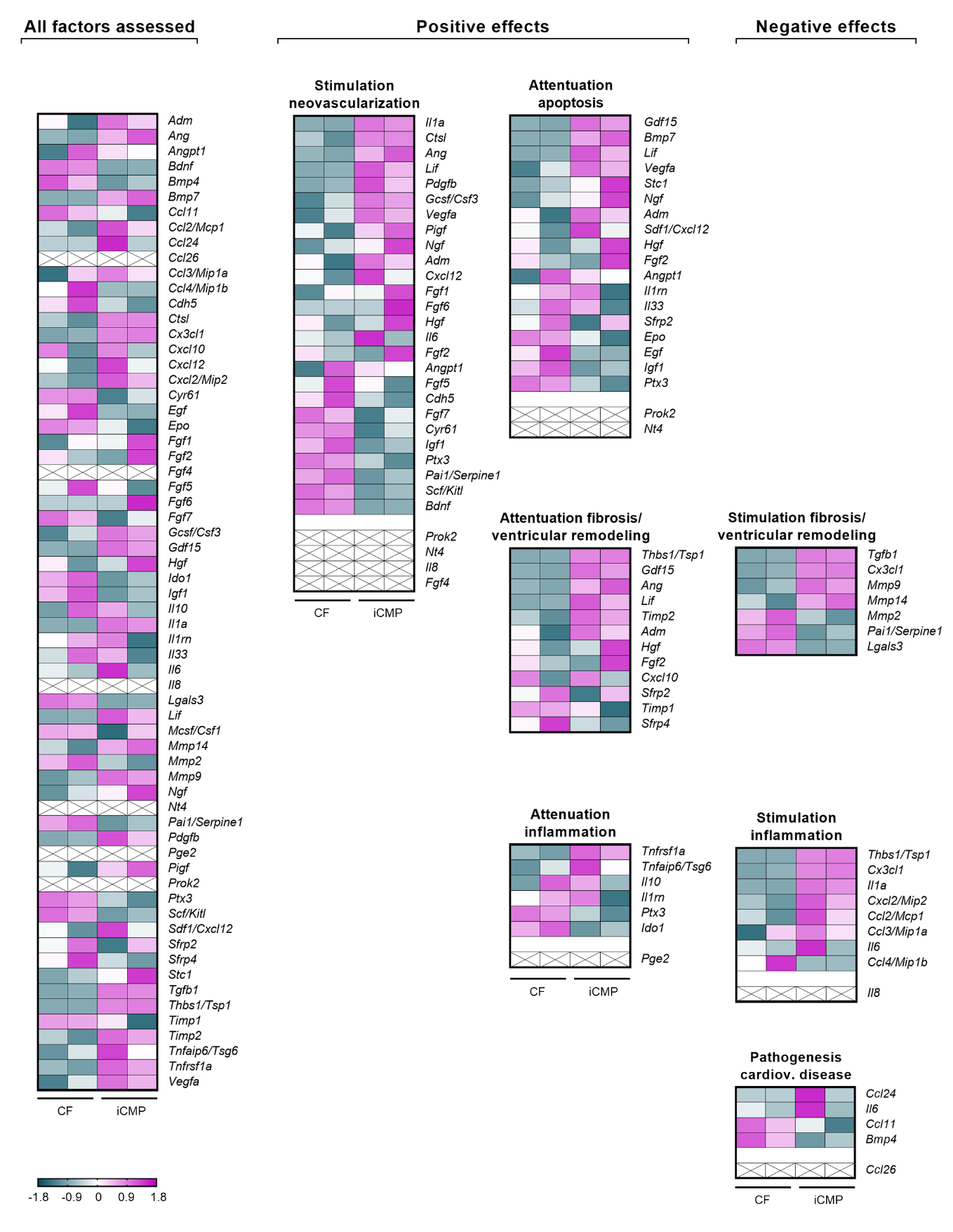


## Fig. S8

**Fig. S8** **Explorative analysis of global transcriptome data for paracrine factors implicated in cardiac repair suggests a cardioprotective secretome for iCMPs.** Gene expression data was generated by RNA sequencing and analyzed for selected genes with a documented positive and/ or negative role in cardiac repair processes, including neovascularization, apoptosis, fibrosis, ventricular remodeling, inflammation, and pathogenesis of cardiovascular disease. Gene selection was based on a comprehensive literature review by Ranganath et. al. [38]. Results are depicted as heatmaps. Heatmap columns represent replicate samples, heatmap rows represent single genes, the Z-score color scale indicates relative gene expression levels for each row (magenta, higher expression, Z-score>0; cyan, lower expression, Z-score<0; cells crossed with an X, gene not detected)

**Table S1 Antibody details**

## Table S1

| Immunogen, conjugate | Host | Company | Catalog no. | Application | Dilution factor |  |
| --- | --- | --- | --- | --- | --- | --- |
| *Primary antibodies (clone)* |  |  |  |  |  |  |
| α-Actinin (EA-53) | Mouse | Sigma-Aldrich | A7811 | IC | 800 |  |
| α-SMA (4A4) | Mouse | Abcam | ab119952 | IC | 200 |  |
| CXCR4 (4G10) | Mouse | Santa Cruz Biotechnology | sc-53534 | IC | 200 |  |
| GATA4 (polyclonal) | Goat | Santa Cruz Biotechnology | sc-1237 | IC | 200 |  |
| GFP (polyclonal) | Chicken | Abcam | ab13970 | IC/IHC; FC | 1,000; 2,000 |  |
| ISL1 (B-1) | Mouse | Santa Cruz Biotechnology | sc-390793 | IC | 200 |  |
| KDR (A-3) | Mouse | Santa Cruz Biotechnology | sc-6251 | IC | 200 |  |
| c-Kit (E-3) | Mouse | Santa Cruz Biotechnology | sc-365504 | IC | 200 |  |
| MEF2C (D80C1) | Rabbit | New England Biolabs | 5030 | IC | 200 |  |
| Myosin (MF-20) | Mouse | Dev. Studies Hybridoma Bank | MF-20 | IC | 200 |  |
| MYOCD (polyclonal) | Goat | Santa Cruz Biotechnology | sc-21561 | IC | 200 |  |
| NKX2-5 (A-3) | Mouse | Santa Cruz Biotechnology | sc-376565 | IC | 200 |  |
| CD31 (polyclonal) | Rabbit | Abcam | ab28364 | IC | 200 |  |
| CD31 (H-3) | Mouse | Santa Cruz Biotechnology | sc-376764 | IHC | 1,600 |  |
| TBX5 (polyclonal) | Goat | Santa Cruz Biotechnology | sc-17866 | IC | 200 |  |
| cTnT (1C11) | Mouse | Abcam | ab8295 | IC/FC | 200 |  |
| cTnI (polyclonal) | Goat | HyTest | 4T21/2 | IHC | 1,000 |  |
| *Secondary antibodies* |  |  |  |  |  |  |
| Chicken, FITC | Donkey | Invitrogen | SA1-72000 | IHC | 250 |  |
| Chicken, Alexa Fluor 488 | Goat | Invitrogen | A11039 | IC | 2,000 |  |
| Goat, Alexa Fluor 647 | Donkey | Invitrogen | A-21447 | IHC | 250 |  |
| Goat, Alexa Fluor 647 | Rabbit | Abcam | AB169347 | IC | 1,000 |  |
| Mouse, Alexa Fluor 555 | Donkey | Invitrogen | A-31570 | IHC | 250 |  |
| Mouse, Alexa Fluor 647 | Goat | New England Biolabs | 4410 | IHC | 1,000 |  |
| Rabbit, Alexa Fluor 647 | Goat | Invitrogen | A-21245 | IC | 1,000 |  |
| Abbreviations: IC, immunocytology; IHC, immunohistology; FC, flow cytometry. | | | | | | |

**Table S2 Reverse transcription-quantitative PCR primer sequences**

## Table S2

| Gene | Forward primer 5' → 3' | Reverse primer 5' → 3' |
| --- | --- | --- |
| *B2m* | TTCTGGTGCTTGTCTCACTGA | CAGTATGTTCGGCTTCCCATTC |
| *Cnn1* | GGCCCAGAAATACGACCATCA | GGTGCCAGTTCTGAGTTGACT |
| *Gata4* | GCCTGTATGTAATGCCTGCG | CATTGCTGGAGTTACCGCTG |
| *Hand2* | TCATGGATCTGCTGGCCAAG | TCTTGTCGTTGCTGCTCACT |
| *Kdr* | GGCGACTATGTTTGCTCTGC | CGCCAATGGTTGTTGTCTGA |
| *Mef2a* | CTAGGACAAGCAGCCCTCAG | GGAGGTGAAATTGGCTCTGACT |
| *Mef2c* | ACGAGGATAATGGATGAGCGT | TGCAATCTCACAGTCGCACA |
| *Mki67* | ACCTGGTCTTAGTTCCGTTGAT | CTTGGTTGGCGTTTCTCCTC |
| *Myl4* | CAAACCCAAGCCTGAAGAGATG | ACCCATGACTGTGCCGTT |
| *Myocd* | AGAATGATGCAGCCTCCCAG | CGGTTCTTACTGTCACCCAAAG |
| *Myod1* | CTGCTCTGATGGCATGATGGAT | CGCTCCACTATGCTGGACAG |
| *Myom1* | GGGCGACACTTACGTTCTCTC | CACCACATCCAAGGGTGCA |
| *Nkx2-5* | CCCAAGTGCTCTCCTGCTTT | CCATCCGTCTCGGCTTTGT |
| *Rpl13a* | TGAAGCCTACCAGAAAGTTTGC | TCCGATAGTGCATCTTGGCC |
| *Scn5a* | GAGATGCTGCTCAAGTGGGT | TCTCGGCAAAGCCTAAGGTG |
| *Tbx5* | ATGAACGTGAACTGTGGCTGA | GGCCAGTCACCTTCACTTTGT |
| *Tnnt2* | CAGAGGAGGCCAACGTAGAAG | CTCCATCGGGGATCTTGGGT |
| *Vim* | GATCAGCTCACCAACGACAAG | GTTCAAGGTCAAGACGTGCC |
| *Vwf* | GTATGGCCCACTACCTCACC | CTGCAACCCTCATTTCCCAC |

**Table S3 Proteins of the secretome (gene ontology term 0005576 “extracellular region”) of iCMPs and CFs as detected by mass spectrometry and their assignment to biological processes and cellular components related to cardiac repair**

## Table S3

| Protein (Gene symbol) | Regulation  of cell death  (GO:  0010941) | | Wound  healing  (GO:  0042060) | | Extracell.  matrix  (GO:  0031012) | | Inflamm.  response  (GO:  0006954) | | Regulation of cytokine production  (GO:  0001817) | | Regulation of cell pop.  proliferat.  (GO:  0042127) | | Regener.  (GO:  0031099) | | Heart  develop.  (GO:  0007507) | | Regulation of angiog.  (GO:  0045765) | | Full name  protein |
| --- | --- | --- | --- | --- | --- | --- | --- | --- | --- | --- | --- | --- | --- | --- | --- | --- | --- | --- | --- |
| ADA10 |  | iCMP |  |  |  | iCMP |  |  |  |  |  | iCMP |  |  |  |  |  |  | Disintegrin and metalloproteinase domain-containing protein 10 |
| AEBP1 |  |  |  |  |  |  |  |  |  |  |  |  |  |  |  |  |  |  | Adipocyte enhancer-binding protein 1 |
| AIMP1 |  |  |  |  |  |  | CF | iCMP |  |  | CF | iCMP |  |  |  |  |  |  | Aminoacyl tRNA synthase complex-interacting multifunctional protein 1 |
| AKR1B3 | CF | iCMP |  |  |  |  |  |  |  |  | CF | iCMP |  |  |  |  |  |  | Aldose reductase |
| ALAD |  |  |  |  |  |  |  |  |  |  |  |  |  |  |  |  |  |  | Delta-aminolevulinic acid dehydratase |
| ALB | CF | iCMP |  |  | CF | iCMP |  |  |  |  |  |  |  |  |  |  |  |  | Serum albumin |
| ALDOA |  |  |  |  |  |  |  |  |  |  |  |  |  |  |  |  |  |  | Fructose-bisphosphate aldolase A |
| ANXA1 | CF | iCMP |  |  | CF | iCMP | CF | iCMP | CF | iCMP | CF | iCMP | CF | iCMP |  |  | CF | iCMP | Annexin A1 |
| ANXA2 |  |  |  |  | CF | iCMP |  |  |  |  | CF | iCMP |  |  |  |  |  |  | Annexin A2 |
| ANXA3 |  |  |  |  |  | iCMP |  |  |  |  |  |  |  | iCMP |  |  |  | iCMP | Annexin A3 |
| ANXA4 |  |  |  |  | CF | iCMP |  |  | CF | iCMP |  |  |  |  |  |  |  |  | Annexin A4 |
| ANXA5 | CF | iCMP | CF | iCMP | CF | iCMP |  |  |  |  |  |  |  |  |  |  |  |  | Annexin A5 |
| ANXA6 |  |  | CF | iCMP | CF | iCMP |  |  |  |  |  |  |  |  |  |  |  |  | Annexin A6 |
| APOE | CF |  |  |  | CF |  |  |  |  |  | CF |  |  |  |  |  |  |  | Apolipoprotein E |
| APP | CF | iCMP |  |  |  |  | CF | iCMP | CF | iCMP | CF | iCMP |  |  |  |  |  |  | Amyloid-beta A4 protein |
| ARHGDIA |  |  |  |  |  |  |  |  |  |  |  |  |  |  |  |  |  |  | Rho GDP-dissociation inhibitor 1 |
| B2M |  |  |  |  |  |  |  |  | CF |  | CF |  |  |  |  |  |  |  | Beta-2-microglobulin |
| BGN |  |  |  |  |  | Bgn |  |  |  |  |  |  |  |  |  |  |  |  | Biglycan |
| C1S1 |  |  |  |  |  |  |  |  |  |  |  |  |  |  |  |  |  |  | Complement C1s-1 subcomponent |
| C3 |  |  | CF |  |  |  | CF |  | CF |  |  |  |  |  |  |  | CF |  | Complement C3 |
| CALR |  |  |  |  | CF | iCMP |  |  |  |  | CF | iCMP |  |  | CF | iCMP |  |  | Calreticulin |
| CALU |  |  |  |  |  |  |  |  |  |  |  |  |  |  |  |  |  |  | Calumenin |
| CAP1 |  |  |  |  |  |  |  |  |  |  |  |  |  |  |  |  |  |  | Adenylyl cyclase-associated protein 1 |
| CAT |  | Cat |  |  |  |  |  |  |  |  |  |  |  |  |  |  |  |  | Catalase |
| CCDC80 |  |  |  |  | CF | iCMP |  |  |  |  |  |  |  |  |  |  |  |  | Coiled-coil domain-containing protein 80 |
| CD109 |  |  |  |  |  |  |  |  |  |  | CF | iCMP |  |  |  |  |  |  | CD109 antigen |
| CFH |  |  | CF |  |  |  | CF |  |  |  |  |  |  |  |  |  |  |  | Complement factor H |
| CFL1 |  |  |  |  |  |  |  |  |  |  |  |  |  |  |  |  |  |  | Cofilin-1 |
| CKB |  |  |  |  |  |  |  |  |  |  |  |  |  |  |  |  |  |  | Creatine kinase B-type |
| CLIC1 |  |  |  |  |  |  |  |  |  |  |  |  |  |  |  |  |  |  | Chloride intracellular channel protein 1 |
| CLU | CF | iCMP |  |  |  |  | CF | iCMP | CF | iCMP | CF | iCMP |  |  |  |  |  |  | Clusterin |
| COL12A1 |  |  |  |  |  | iCMP |  |  |  |  |  |  |  |  |  |  |  |  | Collagen alpha-1(XII) chain |
| COL1A1 |  |  | CF | iCMP | CF | iCMP |  |  |  |  |  |  |  |  |  |  |  |  | Collagen alpha-1(I) chain |
| COL1A2 |  |  |  |  | CF | iCMP |  |  |  |  |  |  |  |  |  |  |  |  | Collagen alpha-2(I) chain |
| COL3A1 |  |  | CF | iCMP | CF | iCMP |  |  |  |  |  |  |  |  | CF | iCMP |  |  | Collagen alpha-1(III) chain |
| COL5A1 |  |  |  | iCMP |  | iCMP |  |  |  |  |  |  |  |  |  | iCMP |  |  | Collagen alpha-1(V) chain |
| COL5A2 |  |  |  |  |  | iCMP |  |  |  |  |  |  |  |  |  |  |  |  | Collagen alpha-2(V) chain |
| COL6A1 |  |  |  |  | CF | iCMP |  |  |  |  |  |  |  |  |  |  |  |  | Collagen alpha-1(VI) chain |
| COPA |  |  |  |  |  |  |  |  |  |  |  |  |  |  |  |  |  |  | Coatomer subunit alpha |
| CP |  |  |  |  |  |  |  |  |  |  |  |  |  |  |  |  |  |  | Ceruloplasmin |
| CPE |  |  |  |  |  |  |  |  |  |  |  |  |  |  | CF | iCMP |  |  | Carboxypeptidase E |
| CST3 | CF | iCMP |  |  | CF | iCMP |  |  |  |  | CF | iCMP |  |  |  |  |  |  | Cystatin-C |
| CTHRC1 |  |  |  |  |  | iCMP |  |  |  |  |  | iCMP |  |  |  |  |  |  | Collagen triple helix repeat-containing protein 1 |
| CTSB | CF | iCMP |  |  | CF | iCMP |  |  |  |  |  |  |  |  |  |  |  |  | Cathepsin B |
| CTSD |  | iCMP |  |  |  | iCMP |  |  |  |  |  |  |  |  |  |  |  |  | Cathepsin D |
| CTSL |  |  |  |  | CF | iCMP |  |  |  |  | CF | iCMP |  |  |  |  |  |  | Cathepsin L1 |
| CTSZ | CF | iCMP |  |  | CF | iCMP |  |  |  |  | CF | iCMP |  |  |  |  |  |  | Cathepsin Z |
| DAG1 |  |  | CF | iCMP | CF | iCMP |  |  |  |  |  |  | CF | iCMP |  |  |  |  | Dystroglycan |
| DBI |  |  |  |  |  |  |  |  |  |  |  |  |  |  |  |  |  |  | Acyl-CoA-binding protein |
| DCN |  |  | CF | iCMP | CF | iCMP |  |  |  |  |  |  |  |  |  |  | CF | iCMP | Decorin |
| DCTN1 |  |  |  |  |  |  |  |  |  |  |  |  |  |  |  |  |  |  | Dynactin subunit 1 |
| DPP7 |  |  |  |  |  |  |  |  |  |  |  |  |  |  |  |  |  |  | Dipeptidyl peptidase 2 |
| DPYSL3 |  |  |  |  |  |  |  |  |  |  |  |  |  |  |  |  |  |  | Dihydropyrimidinase-related protein 3 |
| ECM1 |  |  |  |  | CF | iCMP | CF | iCMP |  |  | CF | iCMP |  |  |  |  | CF | iCMP | Extracellular matrix protein 1 |
| EFEMP1 |  |  |  |  |  | iCMP |  |  |  |  |  | iCMP |  |  |  |  |  |  | EGF-containing fibulin-like extracellular matrix protein 1 |
| EMILIN1 |  |  |  |  |  | iCMP |  |  |  |  |  |  |  |  |  | iCMP |  | iCMP | EMILIN-1 |
| FBLN1 |  |  | CF | iCMP | CF | iCMP |  |  | CF | iCMP | CF | iCMP |  |  |  |  |  |  | Fibulin-1 |
| FBLN2 |  |  |  |  | CF | iCMP |  |  |  |  |  |  |  |  |  |  |  |  | Fibulin-2 |
| FBLN5 |  |  |  |  | CF | iCMP |  |  |  |  | CF | iCMP |  |  |  |  | CF | iCMP | Fibulin-5 |
| FBN1 |  |  |  |  |  | iCMP |  |  |  |  |  |  |  |  |  | iCMP |  |  | Fibrillin-1 |
| FN1 | CF | iCMP | CF | iCMP | CF | iCMP | CF | iCMP | CF | iCMP | CF | iCMP |  |  | CF | iCMP |  |  | Fibronectin |
| GARS |  |  |  |  |  |  |  |  |  |  |  |  |  |  |  |  |  |  | Glycine-tRNA ligase |
| GBA |  | iCMP |  |  |  |  |  |  |  | iCMP |  |  |  |  |  |  |  |  | Lysosomal acid glucosylceramidase |
| GLB1 |  |  |  |  |  |  |  |  |  |  |  |  |  |  |  |  |  |  | Beta-galactosidase |
| GNAS |  |  |  | iCMP |  |  |  |  |  |  |  | iCMP |  |  |  |  |  |  | Guanine nucleotide-binding protein G(s) subunit alpha isoforms XLas |
| GPI1 | CF | iCMP |  |  |  |  |  |  |  |  |  |  |  |  |  |  |  |  | Glucose-6-phosphate isomerase |
| GSN | CF | iCMP |  |  |  |  |  |  |  |  |  |  | CF | iCMP |  |  |  |  | Gelsolin |
| GSTM1 |  |  |  |  |  |  |  |  |  |  |  |  |  |  |  |  |  |  | Glutathione S-transferase Mu 1 |
| GSTM2 |  |  |  |  |  |  |  |  |  |  |  |  |  |  |  |  |  |  | Glutathione S-transferase Mu 2 |
| GSTO1 |  |  |  |  |  | iCMP |  |  |  |  |  |  |  |  |  |  |  |  | Glutathione S-transferase omega-1 |
| HDGF |  |  |  |  |  |  |  |  |  |  |  |  |  |  |  |  |  |  | Hepatoma-derived growth factor |
| HDLBP |  |  |  |  |  |  |  |  |  |  |  |  |  |  |  |  |  |  | Vigilin |
| HEXB |  |  |  |  |  |  |  |  |  |  |  |  |  |  |  |  |  |  | Beta-hexosaminidase subunit beta |
| HMGB1 |  |  |  |  |  |  | CF | iCMP | CF | iCMP | CF | iCMP |  |  |  |  | CF | iCMP | High mobility group protein B1 |
| HMGB2 | CF | iCMP |  |  |  |  | CF | iCMP | CF | iCMP | CF | iCMP |  |  |  |  |  |  | High mobility group protein B2 |
| HNRNPA2B1 |  |  |  |  |  |  |  |  |  |  |  |  |  |  |  |  |  |  | Heterogeneous nuclear ribonucleoproteins A2/B1 |
| HSP90AA1 |  |  |  |  | CF | iCMP |  |  |  |  |  |  |  |  |  |  |  |  | Heat shock protein HSP 90-alpha |
| HSP90AB1 | CF | iCMP |  |  |  |  |  |  | CF | iCMP |  |  |  |  |  |  |  |  | Heat shock protein HSP 90-beta |
| HSP90B1 | CF | iCMP |  |  |  |  |  |  |  |  |  |  |  |  |  |  |  |  | Endoplasmin |
| HSPA4 | CF | iCMP |  |  |  |  |  |  |  |  |  |  |  |  |  |  |  |  | Heat shock protein 4 |
| HSPA5 | CF | iCMP |  |  |  |  |  |  |  |  |  |  |  |  |  |  |  |  | 78 kDa glucose-regulated protein |
| HSPA8 | CF | iCMP |  |  |  |  |  |  |  |  |  |  |  |  |  |  |  |  | Heat shock cognate 71 kDa protein |
| HSPD1 |  | iCMP |  |  |  |  |  |  |  | iCMP |  |  |  |  |  |  |  |  | 60 kDa heat shock protein, mitochondrial |
| IDE |  |  |  |  |  |  |  |  |  |  |  |  |  |  |  |  |  |  | Insulin-degrading enzyme |
| IGFBP7 |  |  |  |  |  | iCMP |  |  |  |  |  |  |  |  |  |  |  |  | Insulin-like growth factor-binding protein 7 |
| ITIH2 |  |  |  |  |  | iCMP |  |  |  |  |  |  |  |  |  |  |  |  | Inter-alpha-trypsin inhibitor heavy chain H2 |
| KARS |  |  |  |  |  |  |  |  |  | iCMP |  | iCMP |  |  |  |  |  |  | Lysine--tRNA ligase |
| LAMA4 |  |  |  |  | CF | iCMP |  |  |  |  |  |  |  |  |  |  |  |  | Laminin subunit alpha-4 |
| LAMA5 |  |  |  |  | CF |  |  |  |  |  | CF |  |  |  |  |  |  |  | Laminin subunit alpha-5 |
| LAMB1 |  |  |  |  | CF | iCMP |  |  |  |  |  |  |  |  |  |  |  |  | Laminin subunit beta-1 |
| LAMC1 |  |  |  |  | CF | iCMP |  |  |  |  |  |  |  |  |  |  |  |  | Laminin subunit gamma-1 |
| LGALS1 |  |  |  |  | CF | iCMP |  |  |  |  |  |  |  |  |  |  |  |  | Galectin-1 |
| LGALS3 |  | iCMP |  |  |  | iCMP |  |  |  |  |  | iCMP |  |  |  |  |  | iCMP | Galectin-3 |
| LGMN |  | iCMP |  |  |  |  |  |  |  |  |  | iCMP |  |  |  |  |  |  | Legumain |
| LOX |  | iCMP |  | iCMP |  | iCMP |  |  |  |  |  |  |  |  |  | iCMP |  |  | Protein-lysine 6-oxidase |
| LTBP1 |  |  |  |  | CF | iCMP |  |  | CF | iCMP |  |  |  |  | CF | iCMP |  |  | Latent-transforming growth factor beta-binding protein 1 |
| MAN2A1 |  |  |  |  |  |  |  |  |  |  |  |  |  |  |  |  |  |  | Alpha-mannosidase 2 |
| MMP2 | CF | iCMP |  |  | CF | iCMP |  |  |  |  | CF | iCMP | CF | iCMP | CF | iCMP |  |  | 72 kDa type IV collagenase |
| MMP3 | CF |  |  |  | CF |  |  |  |  |  |  |  |  |  |  |  |  |  | Stromelysin-1 |
| NAMPT |  |  |  |  |  |  |  | iCMP |  |  |  | iCMP |  |  |  |  |  |  | Nicotinamide phosphoribosyltransferase |
| NID1 |  |  |  |  | CF | iCMP |  |  |  |  |  |  |  |  |  |  |  |  | Nidogen-1 |
| NID2 |  |  |  |  |  | iCMP |  |  |  |  |  |  |  |  |  |  |  |  | Nidogen-2 |
| NUCB1 |  |  |  |  |  |  |  |  |  |  |  |  |  |  |  |  |  |  | Nucleobindin-1 |
| NUCB2 |  |  |  |  |  |  |  |  |  |  |  |  |  |  |  |  |  |  | Nucleobindin-2 |
| NUDCD2 |  |  |  |  |  |  |  |  |  |  |  |  |  |  |  |  |  |  | NudC domain-containing protein 2 |
| OLFML2B |  |  |  |  |  | iCMP |  |  |  |  |  |  |  |  |  |  |  |  | Olfactomedin-like 2B |
| PCOLCE |  |  |  |  |  |  |  |  |  |  |  |  |  |  |  |  |  |  | Procollagen C-endopeptidase enhancer 1 |
| PDAP1 |  |  |  |  |  |  |  |  |  |  |  |  |  |  |  |  |  |  | 28 kDa heat- and acid-stable phosphoprotein |
| PDCD6IP |  |  |  |  |  |  |  |  |  |  |  |  |  |  |  |  |  |  | Programmed cell death 6-interacting protein |
| PDIA3 | CF | iCMP |  |  |  |  |  |  |  |  |  |  |  |  |  |  |  |  | Protein disulfide-isomerase A3 |
| PDIA4 |  |  |  |  |  |  |  |  |  |  |  |  |  |  |  |  |  |  | Protein disulfide-isomerase A4 |
| PDIA6 |  |  | CF | iCMP |  |  |  |  |  |  |  |  |  |  |  |  |  |  | Protein disulfide-isomerase A6 |
| PEBP1 |  |  |  |  |  |  |  |  |  |  |  |  |  |  |  |  |  |  | Phosphatidylethanolamine-binding protein 1 |
| PFN1 |  |  |  |  |  |  |  |  |  |  |  |  |  |  |  |  |  |  | Profilin-1 |
| PKM |  |  |  |  | CF | iCMP |  |  |  |  |  |  | CF | iCMP |  |  | CF | iCMP | Pyruvate kinase PKM |
| PLAA |  |  |  |  |  |  | CF | iCMP |  |  |  |  |  |  |  |  |  |  | Phospholipase A-2-activating protein |
| PLTP |  |  |  |  |  |  |  |  |  |  |  |  |  |  |  |  |  |  | Phospholipid transfer protein |
| PNP | CF | iCMP |  |  |  |  |  |  |  |  | CF | iCMP |  |  |  |  |  |  | Purine nucleoside phosphorylase |
| PPIA |  |  |  |  |  |  |  |  |  |  |  |  |  |  |  |  |  |  | Peptidyl-prolyl cis-trans isomerase A |
| PRKACA |  |  |  |  |  |  |  |  |  |  |  | iCMP |  |  |  |  |  |  | cAMP-dependent protein kinase catalytic subunit alpha |
| PTGIS |  | iCMP |  |  |  |  |  |  |  |  |  |  |  |  |  |  |  | iCMP | Prostacyclin synthase |
| PXDN |  |  |  |  |  | iCMP |  |  |  |  |  |  |  |  |  |  |  |  | Peroxidasin homolog |
| QSOX1 |  |  |  |  |  |  |  |  |  |  |  |  |  |  |  |  |  |  | Sulfhydryl oxidase 1 |
| RBMX |  |  |  |  |  |  |  |  |  |  |  |  |  |  |  |  |  |  | RNA-binding motif protein, X chromosome |
| RBMXL1 |  |  |  |  |  |  |  |  |  |  |  |  |  |  |  |  |  |  | RNA binding motif protein, X-linked-like-1 |
| RNH1 |  |  |  |  |  |  |  |  |  |  |  |  |  |  |  |  | CF | iCMP | Ribonuclease inhibitor |
| RNPEP |  |  |  |  |  |  |  |  |  |  |  |  |  |  |  |  |  |  | Aminopeptidase B |
| RPSA |  |  |  |  | CF | iCMP |  |  |  |  |  |  |  |  |  |  |  |  | 40S ribosomal protein SA |
| S100A10 |  |  |  |  |  | iCMP |  |  |  |  |  |  |  |  |  |  |  |  | Protein S100-A10 |
| SEPT2 |  |  |  |  |  |  |  |  |  |  |  |  |  |  |  |  |  |  | Septin-2 |
| SERPINB1A |  |  |  |  |  | iCMP |  | iCMP |  | iCMP |  |  |  |  |  |  |  |  | Leukocyte elastase inhibitor A |
| SERPINB6A |  |  |  |  | CF | iCMP |  |  |  |  |  |  |  |  |  |  |  |  | Serpin B6 |
| SERPINE1 | CF | iCMP |  |  | CF | iCMP |  |  | CF | iCMP | CF | iCMP |  |  |  |  | CF | iCMP | Plasminogen activator inhibitor 1 |
| SERPINF1 | CF | iCMP |  |  | CF | iCMP |  |  |  |  | CF | iCMP |  |  |  |  | CF | iCMP | Pigment epithelium-derived factor |
| SERPINH1 |  |  |  |  |  | iCMP |  |  |  |  |  |  |  |  |  |  |  |  | Serpin H1 |
| SLC9A3R1 |  | iCMP |  |  |  |  |  |  |  |  |  | iCMP |  |  |  |  |  |  | Na(+)/H(+) exchange regulatory cofactor NHE-RF1 |
| SOD1 | CF |  |  |  |  |  |  |  | CF |  |  |  |  |  |  |  |  |  | Superoxide dismutase [Cu-Zn] |
| SPARC |  |  | CF | iCMP | CF | iCMP |  |  |  |  | CF | iCMP |  |  | CF | iCMP | CF | iCMP | SPARC |
| THBS1 | CF | iCMP |  |  | CF | iCMP | CF | iCMP | CF | iCMP | CF | iCMP |  |  | CF | iCMP | CF | iCMP | Thrombospondin-1 |
| THBS2 |  |  |  |  | CF |  |  |  |  |  |  |  |  |  |  |  | CF |  | Thrombospondin-2 |
| TIMP1 | CF |  | CF |  | CF |  | CF |  |  |  | CF |  |  |  |  |  |  |  | Metalloproteinase inhibitor 1 |
| TIMP2 |  |  |  |  | CF | iCMP |  |  |  |  | CF | iCMP |  |  |  |  |  |  | Metalloproteinase inhibitor 2 |
| TNC |  |  | CF | iCMP | CF | iCMP |  |  |  |  | CF | iCMP | CF | iCMP |  |  |  |  | Tenascin |
| TPT1 |  | iCMP |  |  |  |  |  |  |  |  |  |  |  |  |  |  |  |  | Translationally-controlled tumor protein |
| TXN1 |  | iCMP |  |  |  |  |  |  |  |  |  |  |  |  |  |  |  |  | Thioredoxin |
| VCAM1 |  |  |  |  |  |  |  |  |  |  | CF | iCMP |  |  | CF | iCMP |  |  | Vascular cell adhesion protein 1 |
| VWA5A |  |  |  |  | CF | iCMP |  |  |  |  |  |  |  |  |  |  |  |  | Von Willebrand factor A domain-containing protein 5A |
| XDH | CF | iCMP |  |  |  |  |  |  |  |  | CF | iCMP |  |  |  |  |  |  | Xanthine dehydrogenase/oxidase |
| YBX1 | CF | iCMP |  |  |  |  |  |  |  |  | CF | iCMP |  |  |  |  |  |  | Nuclease-sensitive element-binding protein 1 |

Abbreviations: iCMP, induced cardiomyocyte precursor; CF, cardiac fibroblast. Color code: black, expressed in iCMPs and CFs; magenta, expressed exclusively in iCMPs; grey, expressed exclusively in CFs.
